# Charlson comorbidity health analytics: A population management strategy to identify risk of hospitalizations, repeated hospitalizations, and resultant high cost

**DOI:** 10.1371/journal.pone.0351956

**Published:** 2026-06-29

**Authors:** Mary Charlson, Martin T. Wells, James P. Hollenberg

**Affiliations:** 1 Weill Cornell Medicine Department of Medicine, Weill Cornell Medicine, New York, New York, United States of America; 2 Department of Statistics and Data Science, Cornell University, Ithaca, New York, United States of America; University of Naples Federico II: Universita degli Studi di Napoli Federico II, ITALY

## Abstract

**Background:**

Building on prior development work, the objective of this study of health care utilization of all Weill Cornell Medicine health insurance beneficiaries over a six-year period was to demonstrate the validity of the Charlson Comorbidity Health Analytics (CCHA), a summed weighted measure of 38 chronic conditions in adults and children, that prospectively predict longitudinal risk of hospital admissions, repeated admissions and resultant high cost in populations. The objective of the Charlson Comorbidity Health Analytics (CCHA) is to provide a new foundational framework for population management strategies by identifying the highest risk patients who can then be the focus for interventions designed to reduce unplanned hospitalizations and resultant high costs.

**Methods:**

All 27,190 Weill Cornell Medicine beneficiaries in the years 2016–2021, that is, employees and their dependents, including spouses/partners and their children, were linked across the years in a de-identified way, and CCHA was calculated from claims data. In addition to basic demographics, data included all outpatient and inpatient claims, including payments for each service over each year, excluding pharmacy. While two pharmaceuticals are part of the CCHA (anticoagulants and anti-psychotics), no data about pharmaceuticals was available for this analysis. First, CCHA from each year 2016–2021 was evaluated cross-sectionally as a predictor of that year’s hospitalizations and costs. Second, the CCHA from 2016 beneficiaries who were followed for five years were used to predict longitudinal risk of hospitalizations, repeated hospitalizations, and costs in each of the next five years. Then the CCHA was compared to the CMS Chronic Conditions Warehouse 30 (CCW30) measure. Finally, the CCHA from any given year (2016–2021) was analyzed for its predictive ability over the remaining one to five years of follow-up to predict hospitalizations, repeated hospitalizations, and costs.

**Results:**

Of the total 27,190 beneficiaries over the six years, 55.8% were employees (66.2% women with an average age of 40.9 years), and 25.7% children (average age of 6.1 years). The Charlson Comorbidity Health Analytics (CCHA) score from an index year longitudinally predicts the risk of hospitalizations--including repeated hospitalizations--which drive healthcare costsover six years (p < .01), providing the foundation for interventions in the highest risk patients. Moreover, the 2016 CCHA was a more significant predictor of readmission in 2017–2021 than a 2016 admission. In addition, comorbidity from any index year can be used to predict subsequent admissions and costs; therefore, it works in dynamic populations, like employers and unions that have changes in beneficiaries over time.

**Conclusions and Relevance:**

The Charlson Comorbidity Health Analytics is a method for prospectively identifying the small percent of patients who are at high longitudinal risk for unplanned hospitalizations and high costs. Intervention efforts can then be focused on high-comorbidity patients at high risk [1], with the goal of preventing health deterioration leading to health crises. Comorbidity Health Analytics provides a new foundational framework for population management strategies and specifically for interventions designed to reduce unplanned hospitalizations and thereby reduce costs.

## Introduction

*Comorbidity is “any distinct additional clinical entity that has existed or may occur during the clinical course of a disease that is under study, and that has the potential not only to impact a patient’s prognosis but also to alter their therapeutic plans and outcomes* [1].*”* ***Alvan Feinstein 1970***

Comorbidity is a key determinant of patient prognostic variability and has been extensively evaluated for its role as a confounder for mortality in longitudinal clinical research [2]. A new analytic focus for comorbidity on population management began to emerge from our studies that showed how new criteria could predict hospitalizations and costs in union beneficiaries, Medicaid patients, and Medicare patients [3–7]. Building on mostly cross-sectional and one longitudinal analysis, a new Charlson Comorbidity Health Analytics method (CCHA) that predicts hospitalization, repeated hospitalization, and health care costs longitudinally was developed [3–7]. The analyses demonstrated that a new definition of comorbidity had the potential to provide the foundation for a prospective population management tool [2]. To be successful, a population management strategy has to focus prospectively on the small percent of patients who have the highest persistent longitudinal risk for poor outcomes, repeated hospitalization, and resultant high costs – and this is what the Charlson Comorbidity Health Analytics tool is designed to accomplish.

The Charlson Comorbidity Health Analytics is a method for prospectively identifying the small percent of patients who are at high longitudinal risk for unplanned hospitalizations and high costs. Intervention efforts can then be focused on high-comorbidity patients at high risk [8], with the goal of preventing health deterioration leading to health crises [8,9]. For example, high comorbidity patients cannot be adequately managed in a standard 15-minute primary care visit, followed by multiple specialty referrals, and all too often, urgent/emergent care and hospitalization. Instead, they require longer and more frequent physician time, coaching for self-management, support for social service needs, more frequent follow-ups until stabilization, and support for psychosocial issues and life events [8,10]. This is a method of prospective population management, not a method of risk adjustment for reimbursement.

The original Charlson Comorbidity Index, a weighted measure of 17 specific chronic conditions in adults, was designed to control for the potential confounding impact of prognostically significant comorbid diseases on long-term mortality in observational studies and clinical trials [11]. Prior to the index, many studies simply excluded patients with comorbid disease because of the potential for biased outcomes, but – reducing their generalizability, that is, the patients to whom the results apply [3,12,13]. The original index was developed based on the one-year mortality of 559 medical inpatients and validated based on 10-year mortality from comorbid disease in a population of 685 breast cancer patients [11]. While the populations may seem small, the paper is one of the most widely cited papers in the literature, with over 43,000 citations [14].

If the population management strategy is not focused on the highest risk patients, it will not be successful, and that is what has happened. Over the last few decades, the vast majority of efforts to optimize medical management have focused on patients with multiple chronic diseases believed to be at high risk, defined by different criteria, including multiple chronic diseases, prior hospitalization, and prior high costs. However, these efforts have not reduced hospitalization or costs, nor have they improved outcomes [15–33]. The interventions included disease management, case management, coordinated care, and other interventions, evaluated over some years, have not had the hoped-for success in achieving either objective [25,33–40]. That is not to say that every intervention failed, but many did not achieve their primary objective. In 2016–2017, CMS launched an effort to move away from the millions of combinations of two chronic diseases, which were used to define “multiple chronic conditions.” CMS defined 30 chronic conditions (i.e., Chronic Conditions Warehouse 30 (CCW30) that require ongoing medical attention or limit activities of daily living [41]. The CMS goal was to ultimately reduce cost and utilization, and determined that 30 conditions were drivers of cost [42].

Building on our prior development work, the objective of this study of health care utilization of all 27,190 Weill Cornell Medicine health insurance beneficiaries over a six-year period was to validate the Charlson Comorbidity Health Analytics as a method for prospectively defining longitudinal high risk in populations. This study demonstrates that the Charlson Comorbidity Health Analytics provides a new foundational framework for population management strategies and specifically for interventions designed to improve outcomes, reduce unplanned hospitalizations, and thereby reduce costs.

## B. Methods

The Data Analysis Protocol was submitted to the Weill Cornell Medicine Institutional Review Board (#23–09026543). On October 2, 2023, the WCM IRB determined that this data analysis protocol was non-human subjects research because all data sets from 2016 to 2021 were de-identified and could not be linked back to any individual. Therefore, this precluded the need to secure written or verbal consent.

### B.1 Population

The population consisted of all Weill Cornell health insurance beneficiaries in the years 2016, 2017, 2018, 2019, 2020, and 2021, that is, employees and their dependents, including spouses/partners and their children, for whom one or more claims were filed. Individuals were linked across the years with unique de-identified IDs.

### B.2 Demographic and clinical data

Beneficiary data contained year of birth, gender, and beneficiary status, i.e., employee, spouses/partners, or children. The claims data included all outpatient and inpatient medical, maternity, behavioral health, and other services. Less than 0.01% of claims for each year were processed for service dates more than 12 months prior to the cited year and were excluded from analysis. Pharmacy claims were not included. The Major Diagnostic Category, the servicing providers specialty code, and the line level procedure code, primarily HCPCS level 1 CPT-4, were included. Hospital admission and discharge dates, as well as DRG discharge classification, were noted for those who were admitted to the hospital. Pharmacy claims were not available for this analysis, although anticoagulants and anti-psychotics are included in the CCHA, if available.

Claims data was used to document the total payments for services over each year. In each year, a percent between 4.8% and 6.2% of patients had a claim but no payment. Total amounts paid included inpatient, outpatient, emergency room, and laboratory tests, but not pharmacy costs. The number of admissions was documented along with the specific DRG codes that were assigned.

### B.3 Comorbidity for population management

The Charlson Comorbidity Health Analytics (CCHA) is a predictive population health management system. It is designed as a method of prospectively identifying patients who are likely to incur high costs. In 2014, two papers were published that evaluated the ability of an adapted comorbidity index to predict yearly cost: 1) a total of 186,372 SEIU1199 union beneficiaries (128,182 adults and 58,190 children), and 2) 4,608 Medicaid managed care patients (2,218 adults and 2,396 children) [3,4]. The papers included additional elements: hypertension, depression, warfarin use, and skin ulcers/cellulitis, along with bipolar disease, schizophrenia, drug or alcohol addiction, or the use of anti-psychotic drugs [3,4]. In addition, data from 9,116 patients treated in the Emergency Department who had been enrolled in the Delivery System Reform Incentive Payment, a New York State program for Medicaid beneficiaries, were evaluated along with 11,513 patients from an Accountable Care organization [5]. These data were cross-sectional, with the exception of the SEIU1199 analysis, which included an index year and one year of follow-up [3].

Over the course of these four separate analyses of adults and children who were union, Medicaid, Medicare or ACO beneficiaries, additional conditions were identified and included in predictive models, including inflammatory bowel disease, organ transplants, anti-psychotic drugs, autism, cerebral palsy, cystic fibrosis, intellectual disabilities, Down syndrome, developmental delay, hemophilia, muscular dystrophy, sickle cell, Tay Sachs, and uncontrolled seizures. The new weighted system was evaluated in 21,217 adults and 10,220 children in a regional association of community health centers [43]. It was also evaluated in 158,559 children and 211,168 adults as a predictor of emergency department visits and hospitalizations [6,7]. The Charlson Comorbidity Health Analytics system was built on these analyses to develop predetermined weights for chronic conditions that predict hospitalization, repeated hospitalization, and cost, and it applies to both adults and children.

The weights for the specific chronic conditions and the percent of all Weill Cornell beneficiaries in 2016 with each specific criterion are:

**Table:** 

Comorbidity Health Analytics conditions and weights	Weight	Percent of 2016 Beneficiaries	Percent of 2016 Adults	Percent of 2016 Children
Asthma/Chronic pulmonary disease	1	7.24%	7.08%	7.72%
Cerebrovascular disease	1	1.12%	1.48%	0.03%
Congestive heart failure	1	1.02%	1.35%	0.0%
Dementia	1	0.50%	0.61%	0.0%
Depression	1	6.26%	7.87%	1.37%
Diabetes without end organ damage*	1	5.48%	7.19%	0.29%
Hypertension	1	12.44%	16.45%	0.26%
Inflammatory bowel	1	0.73%	0.94%	0.09%
Myocardial infarction	1	0.23%	0.31%	0.0%
Peptic ulcer disease	1	0.50%	0.66%	0.03%
Peripheral vascular disease	1	1.49%	1.97%	0.06%
Rheumatic disease	1	1.66%	2.14%	0.20%
Warfarin ^#^	1	0.73%	0.96%	0.0%
Cancer, any tumor, leukemia, lymphoma*	2	7.88%	10.24%	0.73%
Cerebral palsy	2	0.02%	0.02%	0.03%
Developmental delay	2	0.73%	0.10%	2.63%
Diabetes with end organ damage*	2	1.01%	1.34%	0.0%
Hemiplegia or paraplegia	2	0.20%	0.23%	0.12%
Mild liver disease	2	1.15%	1.48%	0.18%
Intellectual disabilities	2	0.04%	0.04%	0.03%
Muscular Dystrophy	2	0.01%	0.01%	0.0%
Renal disease	2	1.41%	1.87%	0.03%
Skin ulcer/cellulitis	2	1.73%	1.97%	1.02%
Autism	3	0.33%	0.08%	1.08%
Bipolar	3	0.70%	0.87%	0.18%
Cystic Fibrosis	3	0.03%	0.04%	0.0%
Down syndrome	3	0.04%	0.01%	0.12%
Drug or alcohol	3	0.68%	0.86%	0.15%
Hemophilia	3	0.03%	0.04%	0.0%
Moderate or severe liver disease*	3	0.08%	0.10%	0.03%
Schizophrenia	3	0.17%	0.20%	0.06%
Sickle Cell	3	0.17%	0.17%	0.18%
Tay Sachs	3	0.0%	0.0%	0.0%
Uncontrolled seizures	3	0.20%	0.18%	0.20%
Anti-psychotic drugs	3	--	--	--
AIDS/HIV	6	0.43%	0.54%	0.09%
Metastatic solid tumor*	6	0.46%	0.61%	0.0%
Organ transplant	6	0.17%	0.22%	0.03%

#Pharmaceutical data was not available, but this represents the adverse effects of anti-coagulants.

@ Pharmaceutical data was not available for this population; however, anti-psychotic drugs are part of the index if available.

With three conditions, marked with an asterisk (*) specifically diabetes with end organ damage (weight of 2); moderate to severe liver disease (weight of 3); and metastatic cancer (weight of 6), the weights associated with more severe disease are used exclusively; and in those instances, the less severe associated conditions, diabetes (weight of 1), mild liver disease (weight of 2); and cancer (weight of 2), are set to zero.

### B.4 Chronic conditions warehouse

The Chronic Conditions Warehouse 30 (CCW30) version is comprised of the following conditions: atrial fibrillation, Alzheimer’s, anemia, asthma, benign prostatic hypertrophy, breast cancer, colon cancer, endocrine cancer, lung cancer, prostate cancer, cataract, urologic cancer, congestive heart failure, chronic kidney disease, chronic obstructive pulmonary disease, depression/ bipolar disease, diabetes, glaucoma, hypertension, hip fracture, hyperlipidemia, non-Alzheimer’s dementia, osteoarthritis or rheumatic disease, osteoporosis, Parkinson’s, pneumonia, thyroid disease, and transient ischemic attack/ stroke [42]. Each of the conditions is assigned a weight of one; therefore, CCW30 scores range from 0 to 30 [42].

### B.5 Statistical Methods

The Comorbidity Health Analytics method was evaluated as a predictor of hospital admissions and health care costs; the role of hospital admissions as a mediator of total costs was also analyzed.

**Hospital admissions:** Zero-inflated negative binomial (ZINB) regression models are well-suited for analyzing count data characterized by over-dispersion and an excess of zero values [44]. These models extend the standard negative binomial framework by accounting for the possibility that excess zeros arise from a distinct data-generating process. In this study, a zero-inflated negative binomial model was employed to examine the number of hospital admissions. The model comprises two components: 1) a negative binomial regression to account for the over-dispersed count data, and 2) a probit model to estimate the probability of structural zeros, representing individuals with no likelihood of hospitalization. This dual-model structure allows for more accurate differentiation between patients unlikely to require hospital admissions and those at risk for one or more admissions, thereby enhancing the model’s applicability to complex patterns of health care utilization.

**Total costs:** Two-part models have had widespread application in the evaluation of health care costs [45,46] and consists of two components: 1) a binary outcome model that distinguishes between individuals who did not use any services (zero cost) and 2) those who did (non-zero cost). The first component (zero cost) is estimated using logistic regression. The second component (non-zero cost) is a linear regression model that estimates total health care costs among individuals who incurred any costs. To determine the appropriate functional form for current and prior year (log-transformed) costs, a nonparametric local polynomial smoother was used as an exploratory tool. Since the results suggested that a linear term adequately captured the relationship, as higher-order terms added little to the model’s fit, a linear specification was used.

In the models for the number of hospital admissions and total cost, each of which had two parts, we focused exclusively on the second part in the paper. For the zero-inflated negative binomial models, only the count component modeling the number of hospital admissions is reported. For a total cost we focused exclusively on the second part, which models the log of total cost among those with non-zero expenditures, especially since zero cost only occurred in 3.6%−4.6% of beneficiaries each year between 2016 and 2021.

**Mediation analysis**: Mediation analysis shows that the CCHA relates to hospital admissions and total costs over time. The Baron and Kenny method is a well-established analytical framework used to assess mediation effects in observational and clinical research [47]. This framework is used to evaluate whether the effect of comorbidity (independent variable) on total healthcare cost (dependent variable) is mediated by the number of hospital admissions (mediator). The Baron and Kenny approach involves three sequential regression analyses requiring that: 1) CCHA significantly predicts total cost; 2) CCHA must significantly predict increased admissions; and 3) when CCHA and admissions are included in a regression model to predict cost, the mediator (admissions) must significantly predict cost while controlling for the CCHA. If these criteria are met, the data suggests that hospital admissions may partially or fully explain the observed relationship between comorbidity and cost.

Full mediation is suggested when the direct association between comorbidity and cost becomes statistically non-significant upon inclusion of the mediator. Partial mediation is indicated when the direct effect remains significant but is attenuated. This analytic approach allows for a more nuanced understanding of causal pathways in healthcare utilization and cost, which may inform the design of targeted interventions.

Our mediation analysis aims to understand how the relationship between an independent variable (Charlson Comorbidity Health Analytics, CCHA) and a dependent variable (total cost) is impacted through a mediator variable (the number of admissions) [48]. Generalized Structural Equation Models (GSEMs) offer a framework for estimating and testing these pathways, especially when the data or relationships do not adhere to traditional assumptions of normality or linearity. We propose a direct effect of comorbidity on cost (CCHA → Cost) and an indirect effect of comorbidity on cost through the number of admissions (CCHA → Admissions → Cost). GSEMs integrate link functions, such as the logarithmic function, to model relationships between variables, accommodating non-linear interactions. GSEMs provide a method of estimating these pathways, especially when the data do not adhere to traditional assumptions of normality or linearity. GSEMs integrate link functions, such as the logarithmic function, to model relationships between variables, accommodating non-linear interactions as well as various distributions for outcome variables, including normal or negative binomial distributions.

In the two-part models for total cost and number of hospital admissions, we focused exclusively on the second part, which models the log of total cost among those with non-zero expenditures. For the zero-inflated negative binomial models, only the count component modeling the number of hospital admissions is reported. The results from the first part of the two-part models and the zero-inflation component of the ZINB models are in the supplement.

This analytic approach allows for a more nuanced understanding of causal pathways in healthcare utilization and cost, which may inform the design of targeted interventions.

## C. Results

Of the total 27,190 beneficiaries over the six years, 55.8% were employees, of whom 66.2% were women with an average age of 40.9 ± 14.2 years. Of the remaining 44.2%, 18.5% were spouses/partners, 54.7% were men with an average age of 44.3 ± 12.9 years, and 25.7% were children, who had an average age of 6.1 ± 5.7 years, 48.4% of whom were girls.

### C.1 The temporal composition of the beneficiary population

**Fig 1:**
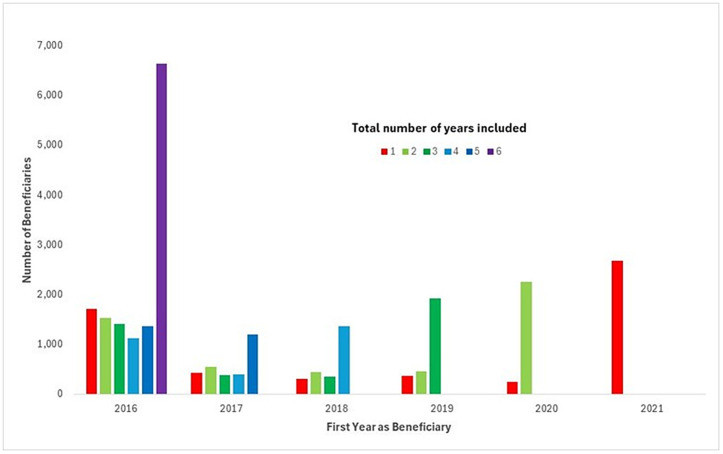
All beneficiaries: first year as beneficiary and total number of years as beneficiary.

In the cross-sectional data, including both adults and children, there were 13,790 individuals from 2016; 14,391 from 2017; 14,886 from 2018; 15,589 from 2019; 16,224 from 2020; and 17,850 from 2021. Fig 1 shows the number of beneficiaries according to the index year, that is, the first year they were included as beneficiaries, compared to the total number of years included.

Over the five years, 11,112 beneficiaries departed Weill Cornell, an average of 2,222 each year, of whom 57.5% were employees (age, 40.1 years); 18.9%, spouses/partners (age, 42.9 years); and 23.6%, children (age, 6.6). Of those who were no longer beneficiaries, only 0.61% (n = 148) employees died over the six years, an average of 25 employees per year and 3 dependents (spouse and children). Over the same years, 14,491 beneficiaries joined Weill Cornell, an average of 2,988 a year; 55.7% employees (age, 37.0 years); 18.2% were spouse/partners (age, 42.9 years) and 26.1% were children (age, 5.2 years). Thus, the average age of employees who departed (40.2 years) differed by only 3.2 years from those who joined (37.0 years).

For adults who were beneficiaries for 6 years, the change in CCHA were small, with 4.7% going from score of 0 to higher scores: increase of 1.4% in those with a score of 1–2; a 1.7% in those with a score of 3–4; an increase of 1.0% in those with a score of 5–7 and an increase of 0.6% in those with a score ≥8.

The first two sections of the results, C1 and C2, analyze cross-sectional data from the years 2016–2021. The third section, C3, focuses on a longitudinal analysis of beneficiaries from 2016 (the index year), who were continuously followed over the subsequent five years. The fourth section, C4, presents a lagged analysis, focused on beneficiaries with 1–5 years of subsequent follow-up after an index year, taking data from the index years, whenever they occurred, to predict hospitalizations, repeated hospitalizations, and costs over the following 1–5 years. Data from 2021 were excluded from this analysis due to the lack of follow-up beyond that year. This analysis illustrates how the CCHA would work in populations as they acquire new beneficiaries over time.

### C.2 Cross-sectional relationship between comorbidity and costs in 2016

**Fig 2:**
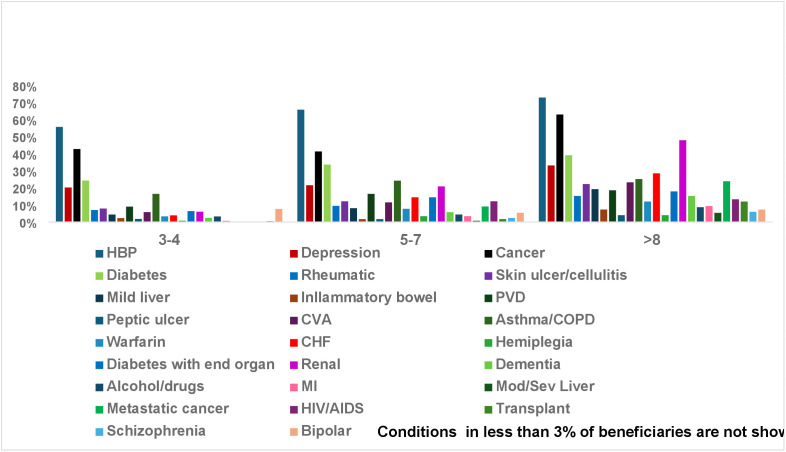
Adults from 2016 with higher comorbidity (>3) have many different conditions.

The cross-sectional relationship of comorbidity, costs, and hospitalizations is explored first for 2016 alone and then for each year between 2017 and 2021. To illustrate that higher comorbidity consists of many different conditions, Fig 2 shows the 12.1% of adults from 2016 who had a comorbidity of >3, that is, scores of 3–4, 5–7, and ≥8 or more, according to the conditions that constituted ≥3% of each comorbidity group. The figure focuses on adults since only 1.6% of children had comorbidity ≥3.

**Fig 3:**
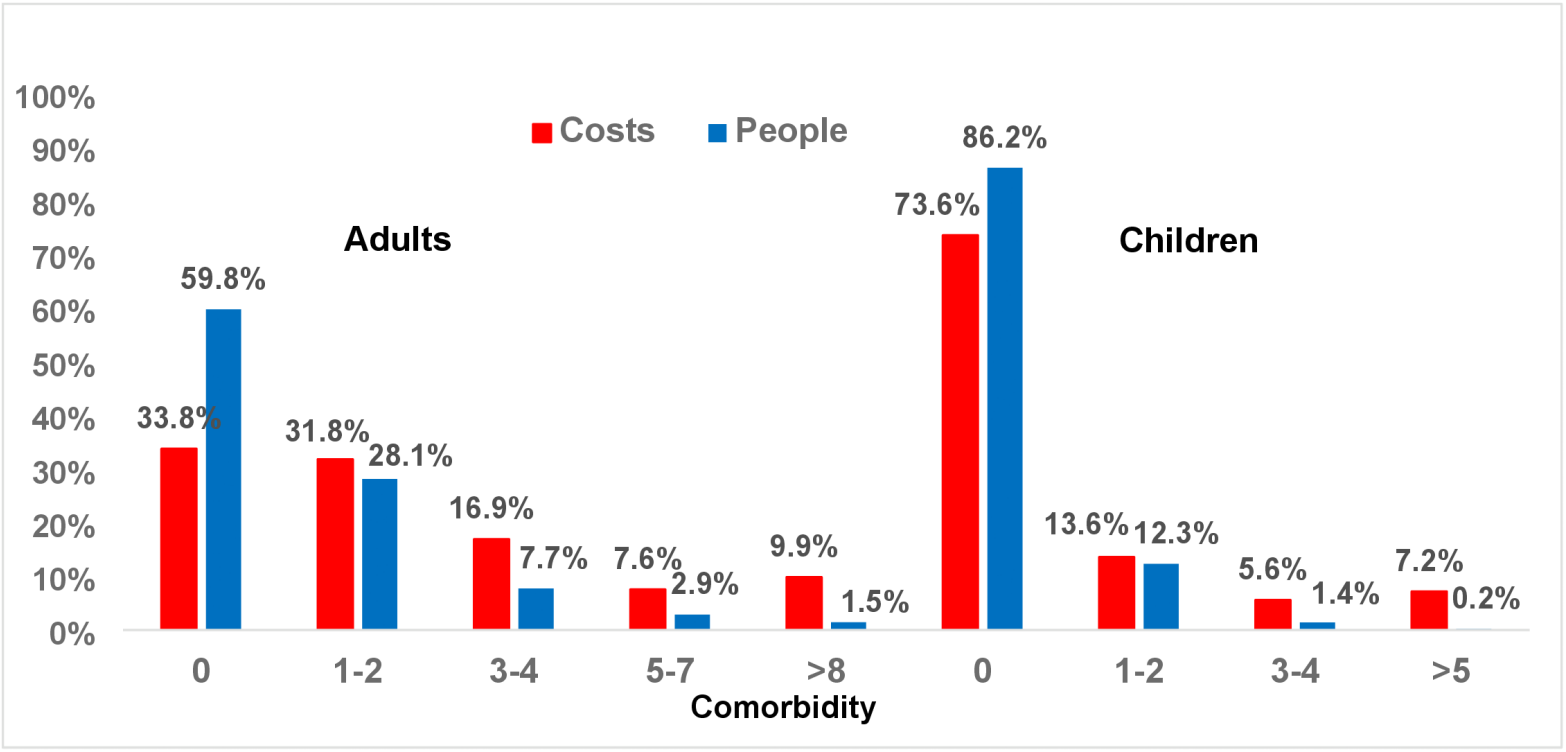
Comorbidity in 2016: Percent of people and costs.

Fig 3 shows the 13,790 beneficiaries in 2016, and controlling for age and gender, comorbidity (CCHA16) significantly predicted yearly cost (P < .01; R^2^ = .11); 75% (10,370) were adults with a total cost of $74.2 million, and 25% (3,420) were children with a total cost of $13.5 million. Adults with a comorbidity score of ≥5 constituted 4.3% of the population and had 17.5% of costs, while 0.2% of children with a comorbidity score of ≥5 had 7.2% of costs. Yearly cost rose steeply with comorbidity from a yearly cost of $3,835 for those with a score of 0; $7,629,1–2; $15,675, 3–4; $18,824,5–7; and $53,189 for those with scores ≥8. Among adults controlling for age and gender, comorbidity predicted cost in adults (P < .01, R^2^ = 0.08) and in children (P < .01; R^2^ = 0.12) (data in S1 Table. Predictors of log-transformed total costs in 2016 for adults and children with non-zero expenditures).

As comorbidity increased, the percent of adults with admissions increased from 1.2% with a score of 0; to 4.7% with a score of 1–2; 15%, with a score of 3–4; 32% with a scores of 5–7; and 61% admitted, with a score ≥8 – dramatic differences in admission rates (*P* < .01). Hospital admissions were strongly associated with annual healthcare costs. Unadjusted costs increased substantially with the number of admissions, rising from $3,898 among individuals with no admissions to $28,436 with one admission, $70,153 with two admissions, and $91,911 with three or more admissions. Among adults in 2016, hospital admissions increased significantly with higher comorbidity scores, after adjusting for age and gender (*P* < .01); (data in S2 Table. Predictors for the number of hospital admissions of adult and child admissions in 2016 from zero-inflated negative binomial regression). In 2016, 2.5% (259) of adult beneficiaries had obstetric admissions in 2016, with an average cost of $28,925.

In 2016, 8.7% (298 of 3,420) children were hospitalized. Among these hospitalized children, 73.5% or 219 were newborns, including both well and sick newborns. Notably, sick newborns incurred costs that were 4.8 times higher than those of well newborns ($29,807 vs. $6,162; *P* < .01). Among the remaining 3,198 children aged over one year (92% of the pediatric population), the hospitalization rate was 2.5%. Although most admitted children had a comorbidity score of 0, hospital admissions in this group still rose significantly with increasing comorbidity, controlling for age and gender (*P* < .01); (data in S2 Table. Predictors for the number of hospital admissions of adult and child admissions in 2016 from zero-inflated negative binomial regression).

The 2016 comorbidity data are presented separately, as they are used subsequently to predict hospital admissions and healthcare costs (Section C4) over the subsequent five years (2017–2021) among beneficiaries who remained continuously enrolled. An additional analysis in Section C.5 examines costs by comorbidity beginning from any index year (i.e., the first year of enrollment), with lagged analyses assessing admissions and costs in the following years.

### C.3 Cross-sectional relationship between comorbidity, costs, and admissions: 2017–2021

Between 2017 and 2021, about 75% of beneficiaries were adults and 25% children. The percent of adults and children with different chronic conditions was not significantly different between 2016 and 2017–2021. Between 2017 and 2021, there were 1,416 pregnancies, each with expected hospital admissions, resulting in an average obstetric yearly cost of $35,432 per person, accounting for 12.05% of total adult costs across the years 2017–2021. For these figures and subsequently, the obstetric costs and admissions are excluded from adult analysis.

**Fig 4:**
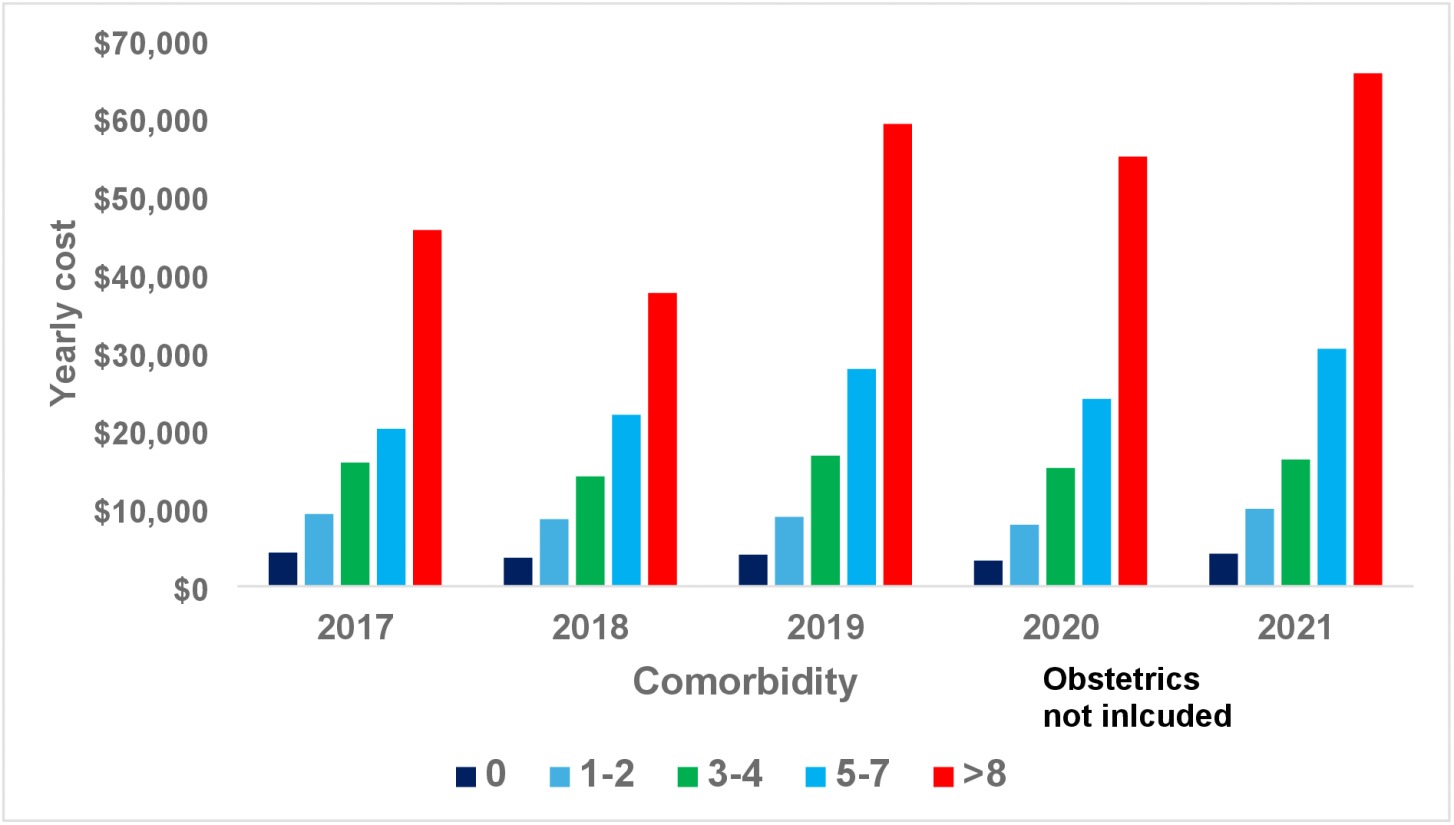
Cross-sectional yearly costs 2017-2021 for adults according to comorbidity.

**Costs for Adults:** Fig 4 shows that costs for adults rose on average across all years 2017–2021 from $3,900 in those with no comorbidity, to $8,900 in those with 1–2; to $15,600 with 3–4; to $25,000 in those with 5–7; and to $53,800 in those with ≥8.

Over the five-year period from 2017 to 2021, adults without comorbidity (60% of the population) accounted for 31.8% of total healthcare (non-obstetric) costs. Adults with a comorbidity score of 1–2 comprised 28% of the population and accounted for 34% of costs. As comorbidity increased, cost concentration became more pronounced: 7.5% of adults with a score of 3–4 incurred 16% of costs; 2.9% with a score of 5–7 accounted for 9.8% of costs; and 1.2% with a comorbidity score ≥8 accounted for 8.4% of total costs across all five years. Controlling for age and gender, comorbidity significantly predicted total cost for adults in 2017 (P < .01; R^2^ = .11); 2018 (P < .01; R^2^ = .097); 2019 (P < .01; R^2^ = .12); 2020 (P < .01; R^2^ = .13); 2021 (P < .01; R^2^ = .14) (data in S3 Table. Predictors of log_10_ total medical and surgical cross-sectional costs for adults in each year 2017–2021). In addition, comorbidity did predict zero total medical and surgical costs in all years 2017–2021, P < .01 for all; (data in S4 Table. Predictors of zero total medical and surgical cross-sectional costs for adults for each year 2017–2021. Two-part regression that models zero cost in 2017–2021). Controlling for age and gender, comorbidity predicted medical costs alone from 2017 to 2021 in a similar way (P < .01 for 2017–2021) (data in S5 Table. Predictors of log total medical cross-sectional costs for adults in each year 2017–2021). Similarly, comorbidity predicted zero medical costs for 2017–2021, P < . 01 for all data in the S6 Table. (Predictors of zero medical (only) costs for adults cross-sectionally for each year 2017–2021. Two-part regression for zero adult total medical costs (only) in 2017–2021).

**Fig 5:**
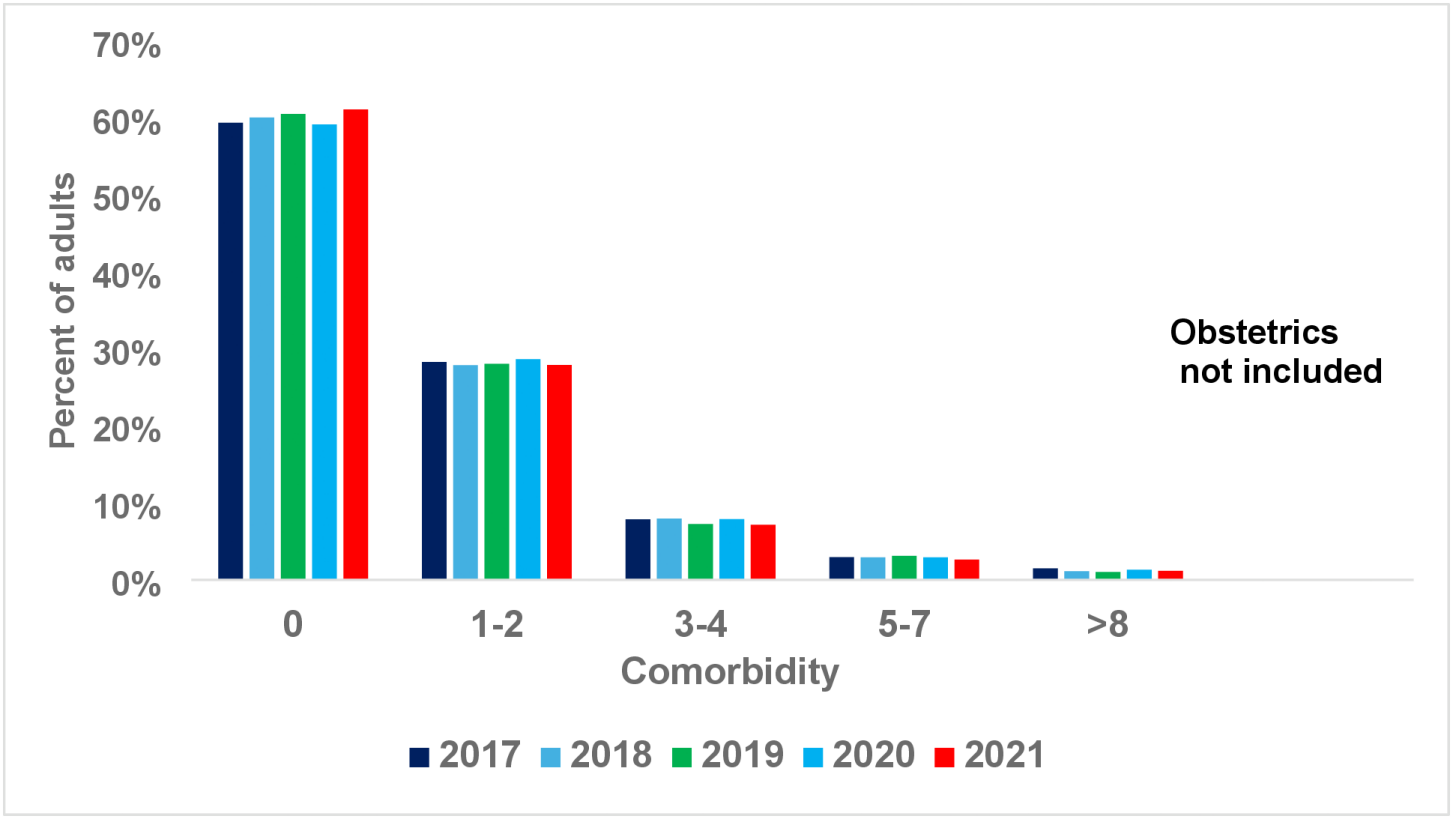
Percent of adults according to comorbidity in the years 2017-2021.

Fig 5 shows the percent of adults according to comorbidity in 2017–2021; about 60% had scores of 0; 28% scores of 1–2; 8% scores of 3–4; 3% scores of 5–7; and 1% scores of ≥8.

**Costs for Children:** Between 2017 and 2021, there were 1,396 newborns with an average cost of $21,642, each with expected hospital admissions; however, sick newborns excluding premature infants had 3.5 times the cost of normal newborns ($37,110 vs $9,360, P < .01). These costs related to newborn care or related-hospital admissions have not been included in the subsequent evaluations of the impact of comorbidity in children.

**Fig 6:**
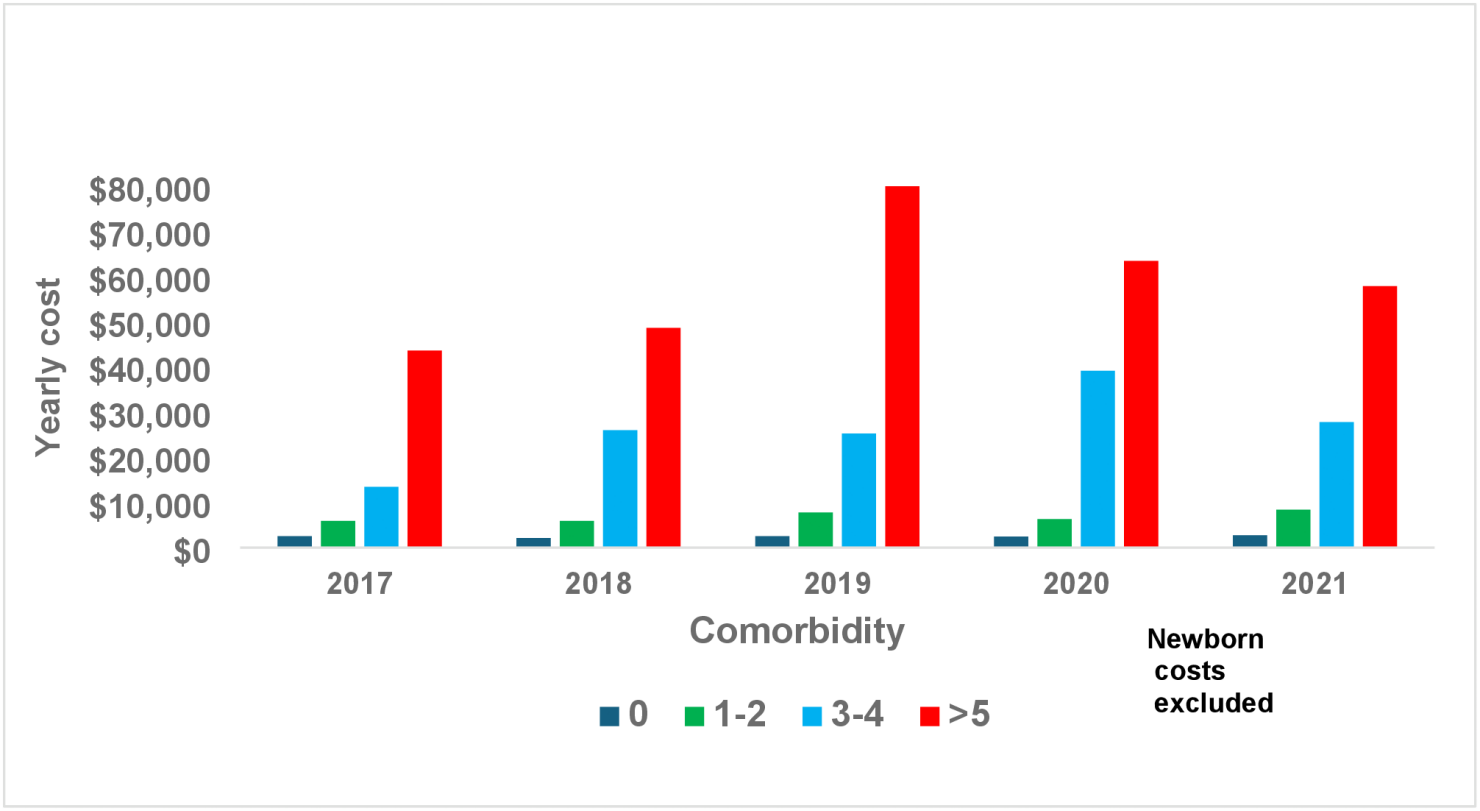
Cross-sectional yearly costs for children according to comorbidity, 2017-2021.

**Fig 7:**
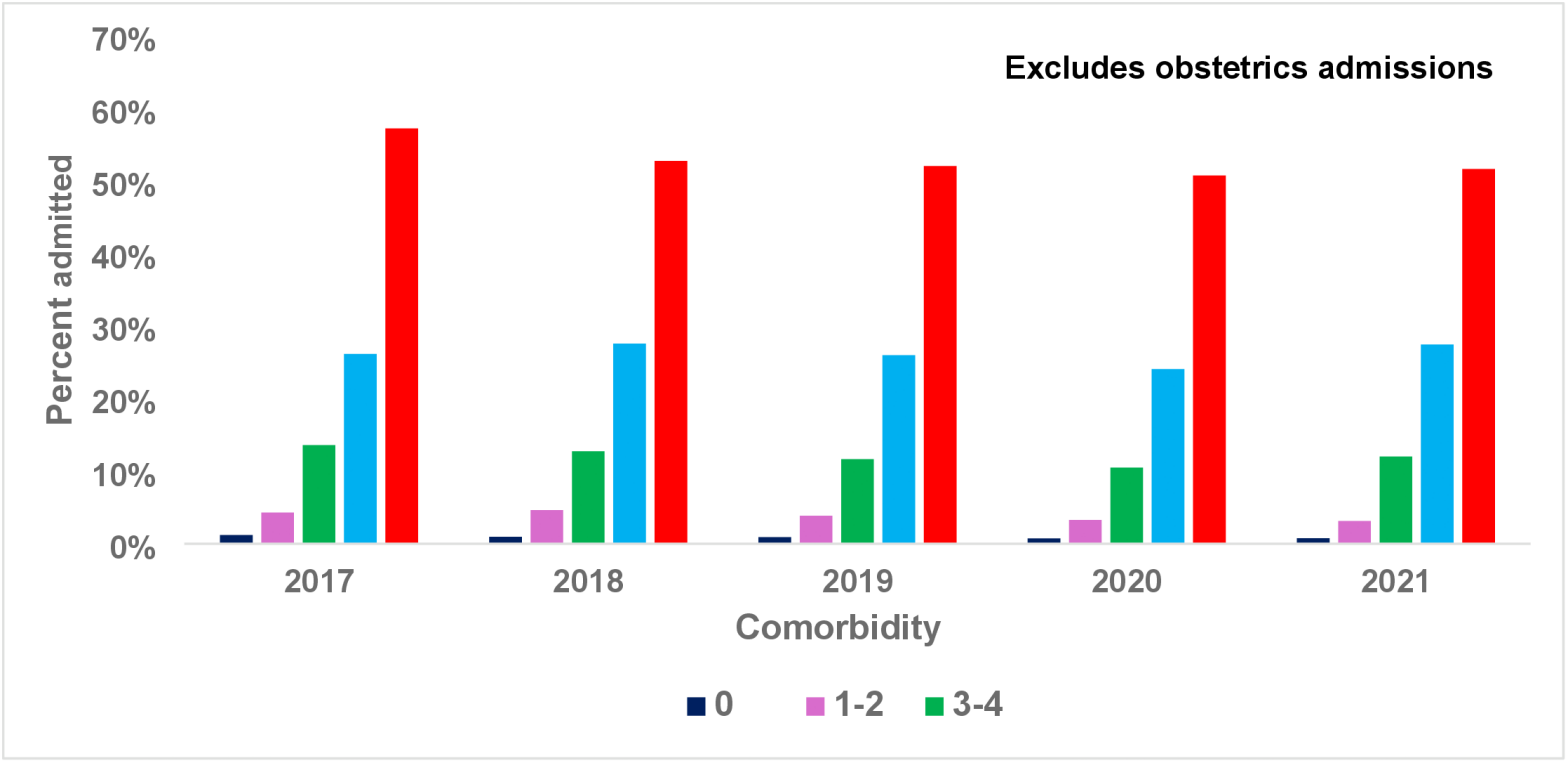
Percent of adults admitted for medical or surgical reasons during 2017-2021 according to comorbidity.

Fig 6 shows yearly costs and comorbidity for children. Overall, 85% of the children had no comorbidity; 13% had a comorbidity of 1–2; the remaining 1.8% of children had a comorbidity of ≥3. Children with comorbidity ≥3 had 15% of the total costs for children. In 2019, there were only 3 children with comorbidity ≥5 who had annual costs ≥ $80,000, so the yearly cost is truncated in the figure (Fig 7).

After adjusting for age and gender, comorbidity was a significant predictor of total healthcare costs for children in each year: 2017 (*P* < .01; R² = 0.12), 2018 (*P* < .01; R² = 0.10), 2019 (*P* < .011; R² = 0.13), 2020 (*P* < .01; R² = 0.08), and 2021 (*P* < .01; R² = 0.09) (data in S7 Table. Predictors of log total cross-sectional costs for children in each year 2017–2021, eliminating newborns). Comorbidity also predicted zero costs for children in 2019 and 2021, P < .01 (data in S8 Table. Predictors of zero cross-sectional costs for children for each year 2017–2021, eliminating newborns. Two-part regression model of zero child total costs that were zero in 2017–2021).

**Adult admissions and costs:** Fig 7 shows the percent of adults admitted each year for medical or surgical reasons, excluding obstetric and psychiatric admissions. Only 0.054% of admissions over 2017–2021 were for psychiatric reasons, with an average cost of $103,097.

The percent of adults admitted to the hospital in each year from 2017 through 2021 increased in a stepwise manner with rising comorbidity scores from 1% with comorbidity scores of 0; 3–5% for those with scores of 1–2; 9%−16% for those with 3–4; 21%−35% for those with 5–7; and 48%−64% for those with comorbidity scores ≥8. Controlling for age and gender, comorbidity was a consistent and independent predictor of adult hospital admissions across all five years (*P* < .01); (data in S9 Table. Predictors of adult admissions cross-sectionally 2017–2021, excluding obstetrics).

During 2017–2021, medical admissions accounted for 55.6% of admissions, while surgical admissions consistently accounted for approximately 32.5% of all hospitalizations. Medical admissions involved diagnoses such as myocardial infarction, cerebrovascular accident, gastrointestinal bleeding, congestive heart failure, and chronic pulmonary disease. Each year, approximately 11.8% of patients had both medical and surgical admissions, with the exception of 2021 during the COVID-19 pandemic, when this overlap rose markedly to 30%. Controlling for age, gender, and comorbidity, costs rose steeply with admissions from $5,000-$6,400 per year with no admissions; to $39,000-$49,000 for those with 1 admission; to $48,000-$82,000 for those with 2 admissions; and to $77,000-$114,000 for those with ≥3 admissions. Total costs each year from 2017–2021 for patients with a surgical admission were 1.5–2 times higher than those for patients with a medical admission.

Over the five years, there were 131 adults, with an average age of 67 ± 19 years, who were admitted more than 4 times for a total of 642 admissions with a total average cost of $205,567 per patient. Notably, 60% had a comorbidity score ≥ 8, and 30% had a score between 5 and 7. This group had a wide variety of conditions: cancer, diabetes, diabetes with end-organ damage, moderate to severe renal impairment, chronic obstructive pulmonary disease, congestive heart failure, peripheral vascular disease, and depression, among other conditions.

**Children admissions and costs**: Between 2017 and 2021, there were 1,287 newborns with an average cost of $20,981. Of the newborns, 58.1% were normal newborns with an average cost of $9.360, and 42.6% were classified as sick, with an average cost of $37,220. These newborns are not included in the rest of the analysis of children in 2017–2021. On average, only 3.3% of babies < 1 year who were not newborns were admitted each year, with a cost of $90,438 per admission, accounting for 37.4% of the total cost for children that age. Overall, 1.7% of children older than 1 year were admitted each year between 2017 and 2021, with an average cost per admission of $43,438, and their admissions accounted for 28.1% of the total cost of children >1 year of age.

**Fig 8:**
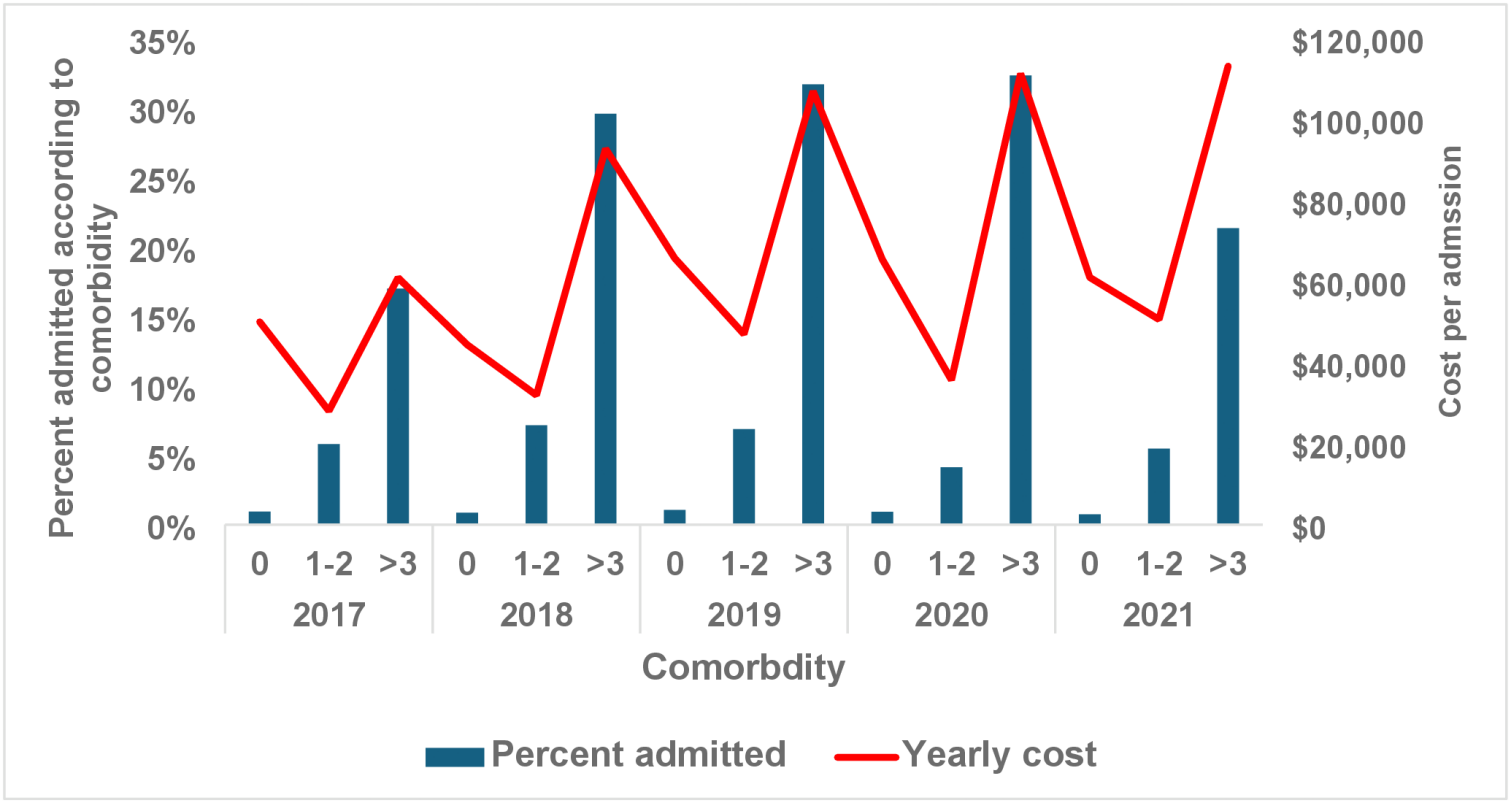
Percent of children admitted by comorbidity and average cost per admission 2017-2021.

Fig 8 shows the percent of children admitted according to comorbidity and the average costs of hospitalization over the five years. Considering all children, excluding newborns, less than 1% of those with a comorbidity score of 0 were admitted, while 4–8% of those with a comorbidity score of 1–2, and 17% to 32% of those with a comorbidity score ≥3 were admitted. The average yearly cost for the 150 admitted children with a comorbidity score of 0 was $58,185; $39,663 for the 152 with a score of 1–2, and $101,300 for the 92 with a comorbidity score ≥3. Across all five years (2017–2021), comorbidity was a significant independent predictor of hospital admission risk for children (*P* < .01; data in S10 Table. Predictors of child admissions in 2017–2021 cross-sectionally, excluding newborns).

Over the five years, there were 21 children, with an average age of 10.7 years, all but three of whom had a comorbidity score of 3 or more, who were admitted more than 4 times for a total of 135 admissions, with a total cost of $313,276 per patient. This small group had a wide variety of conditions: s/p transplant, cancer, rheumatic disease, moderate to severe renal disease, asthma, schizophrenia, bipolar disease, and depression, among other conditions.

Thus, when evaluated cross-sectionally, in adults and children, comorbidity was a significant concurrent predictor of hospitalizations, repeated hospitalizations, and healthcare costs.

### C.4 Comorbidity from one year (2016) predicts subsequent costs, hospitalizations, and repeated hospitalizations over the next five years

To be most useful as a predictor, comorbidity from one year would have to be shown to drive subsequent hospitalizations and costs over multiple years. To evaluate this, comorbidity from 2016 beneficiaries was used to predict their longitudinal risk, that is, their subsequent admissions and costs over the next five years. Over five years, among all adult and child beneficiaries, excluding data from newborn and obstetric beneficiaries, the 58.7% of 2016 beneficiaries with a comorbidity score of zero had average yearly costs of $5,813; increasing to $10,036 among the 28.6% with a score of 1–2; to $13,815 for the 8.6% with a score of 3–4; to $17,945 among the 3.03% with a score of 5–7; and to $28,810 among the 1.1% with a score ≥8 (P < .01).

### 2016 Comorbidity predicts costs over the next five years

**Fig 9:**
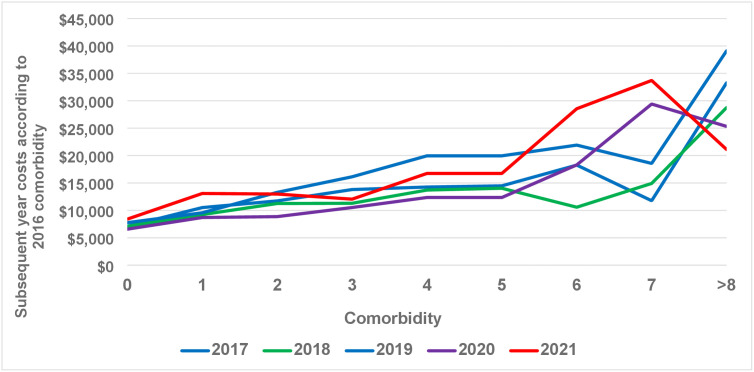
Adults: Comorbidity from 2016 predicting subsequent year costs.

Since the majority of total costs and hospitalizations occur among adults, Fig 9 shows yearly costs for adults over the five-year period from 2017 to 2021, stratified by 2016 comorbidity. Overall, 49.2% of adults from the 2016 had a score of 0; 19.9% a score of 1; 14.0%, a score of 2; 8.0%, a score of 3; 3.3% a score of 4; 2.1%, a score of 5;1.3% a score of 6; 0.7%, a score of 7; and 1.4% a score of ≥8.

Comorbidity measured in 2016 was a significant predictor of healthcare costs in each of the following five years, even after adjusting for age and gender (all *P* < .01; see Table 1). Admission in 2016 significantly predicted total cost only in 2019 (P < .01).

**Table 1:** Longitudinal analysis of 2016 Comorbidity in adults as a predictor of subsequent total costs over the subsequent five years (2017-2021) for beneficiaries covered over the whole five years.

	Total Cost	Total Cost	Total Cost	Total Cost	Total Cost
	2017	2018	2019	2020	2021
					
CCHA2016	.082 ± .006***	.071 ± .007***	.075 ± .007***	.076 ± .007***	.064 ± .007***
					
2016 Admission	.038 ± .028	.060 ± .032*	.059 ± .030**	.030 ± .031	−.037 ± .032
					
R-squared	.060	.046	.058	.062	.047

*** p < 0.01, ** p < 0.05, * p < 0.1 Controlling for age and gender, which are significant at P<.01 in each year 2017-2021.

Standard error for regression coefficients are shown.

### 2016 Comorbidity predicts admissions over the next five years

**Fig 10:**
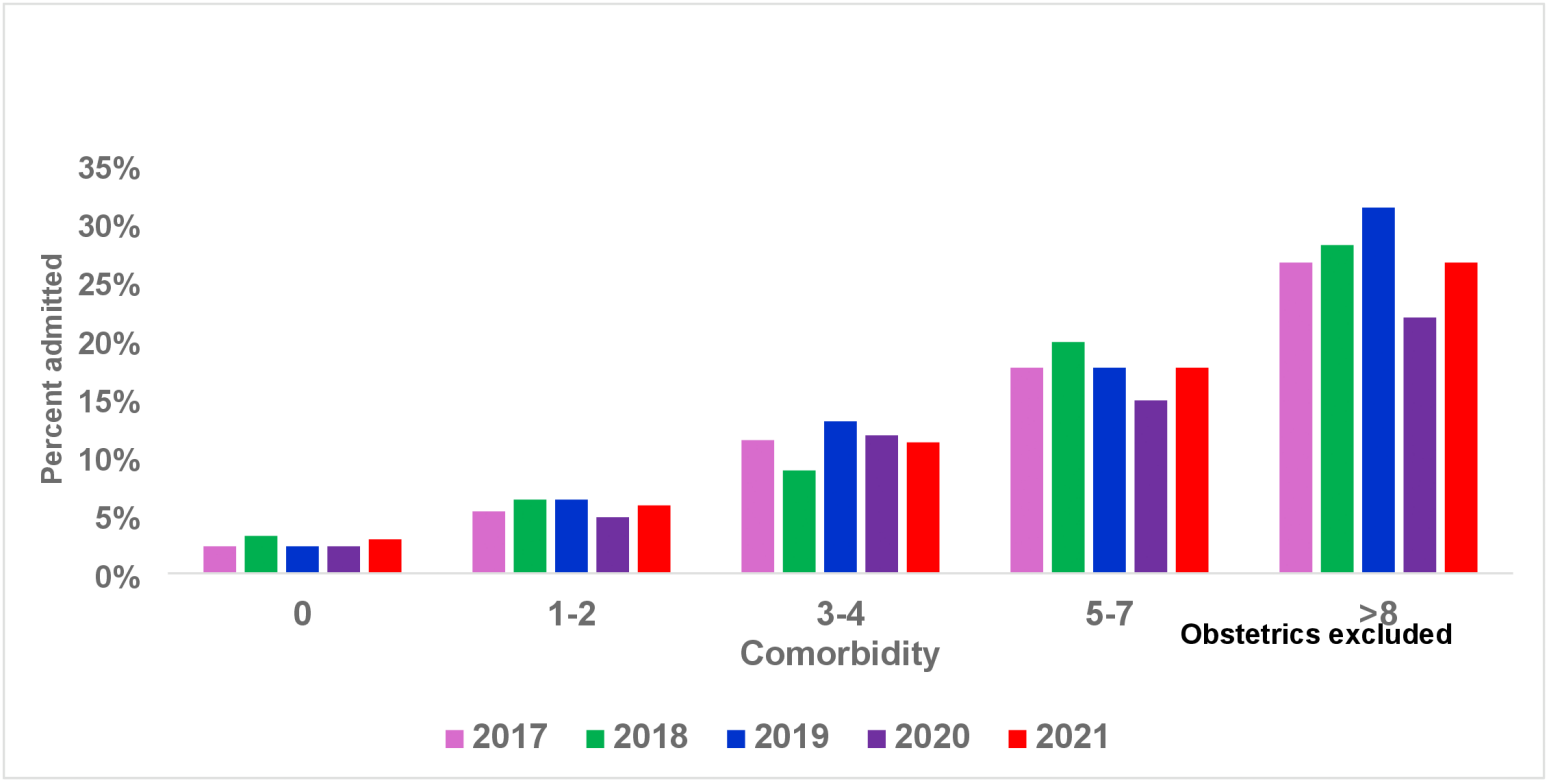
Percent of adults admitted in each year 2017-2021 according to 2016 comorbidity.

Yearly healthcare costs from 2017–2021 are closely tied to the number of hospital admissions each year, and admissions over the five years are significantly predicted by 2016 comorbidity (all *P* < .01; see Table 2), but 2016 admission only predicted admissions for 2017 (P < .01). Table 3 shows that neither 2016 comorbidity nor 2016 admissions predicted zero admissions in 2017–2021. Fig 10 shows that 2016 comorbidity predicts hospital admissions from 2017 to 2021.

Comorbidity assessed in 2016 remained a significant predictor of increased hospitalizations over the subsequent years (all *P* < .01; see Table 2).

**Table 2:** Longitudinal analysis of 2016 Comorbidity in adults as a predictor of subsequent total admissions over the subsequent five years (2017-2021) for beneficiaries covered over the whole five years.

	Total admission	Total admission	Total admission	Total admission	Total admission
	2017	2018	2019	2020	2021
					
					
CCHA 2016	.183 ± −.031***	.223 ± .031***	.215 ± .032***	.205 ± .036***	.178 ± .032***
					
2016 Admission	.379 ± −.111***	.079 ± .124	.238 ± .126*	−.061 ± .151	−.181 ± −.155

*** p < 0.01, ** p < 0.05, * p < 0.1

Controlling for age which is significant in year each 2017–2021 at p < .01; and gender which is significant at p < .01 in 2017 and 2018, and p < .05 in 2019 and 2021. Standard error for regression coefficients are shown.

**Table 3:** Longitudinal analysis of 2016 Comorbidity in adults as a predictor of zero subsequent total admissions over the subsequent five years (2017-2021) for beneficiaries covered over the whole five years. Zero-inflated negative binomial models for adults with zero admissions.

	Zero admissions	Zero admissions	Zero admissions	Zero admissions	Zero admissions
	**2017**	**2018**	**2019**	**2020**	**2021**
					
Admit 2016	−15.6 ± 545	−15.5 ± 1294	−12.8 ± 485	−11.6 ± 426	−14.1 ± 408
CCHA 2016	---	---	---	---	---
					
					
Observations	10,369	10,369	10,369	10,369	10,369
					
					

Controlling for age and gender, which are significant at P<.01 in each year 2018-2021. Standard error for regression coefficients are shown.

### Admissions in 2016 did not reliably predict subsequent admissions over the next five years

Hospital admissions in 2016 were ***not*** reliable predictors of subsequent admissions in 2017–2021, with only marginal significance observed in 2017 (see Table 2). Overall, 85.8% of individuals admitted in 2016 were not hospitalized again in any of the following years, 2017–2021.

**Fig 11:**
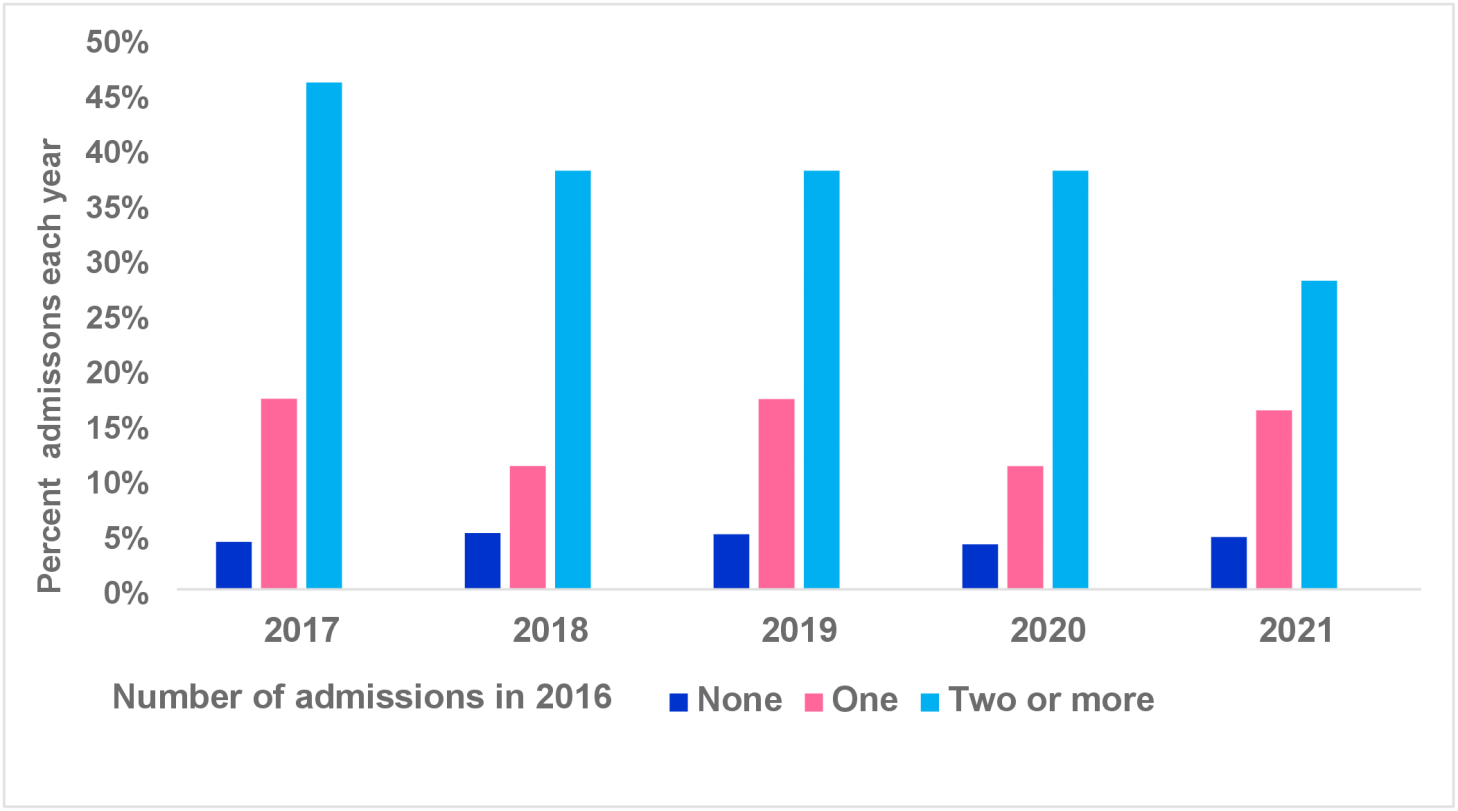
Percent of adults admitted in 2017-2021 according to the number of admissions in 2016.

Fig 11 shows the percent of adults admitted from 2017 to 2021, according to 2016 admissions. It is important to note that overall, 85.8% of adults hospitalized in 2016 were not admitted in any of the following years, 2017–2021. About 4–5% of those without a 2016 admission were admitted subsequently, increasing to 11%−17% of those with one 2016 admission; and to 28% to 46% of those with two or more 2016 admissions.

**Fig 12:**
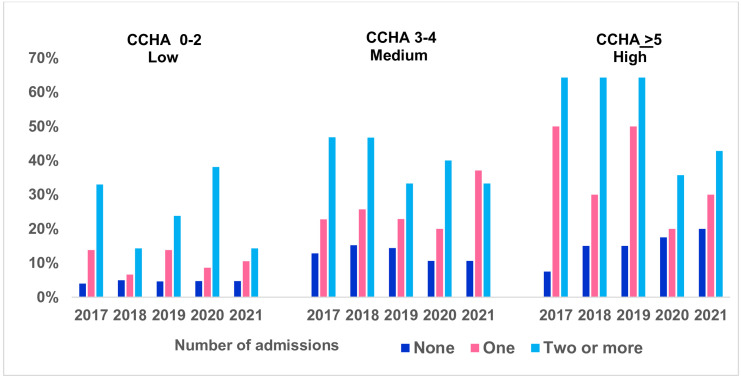
Percent of adults admitted in 2017-2021 according to the number of admissions in 2016.

As shown in Fig 12, although a slightly higher percent of those admitted in 2016 experienced at least one admission between 2017 and 2021 than those who were not admitted, comorbidity emerged as the primary driver of readmissions (all *P* < .01; see Table 2).

In the aggregate, among adults who were admitted in 2016, there were 377 subsequent **total** admissions over the next five years (2017–2021); of those, 31.0% had CCHA scores of ≥8 and 28.1% had scores of 5–7. Thus, of 5.5% of adults with a comorbidity score >5 who were admitted in 2016, 59.1% were readmitted. These findings indicate that comorbidity is a stronger and more consistent predictor of readmission than prior hospitalization status alone (all *P* < .01; see Table 2).

**Fig 13:**
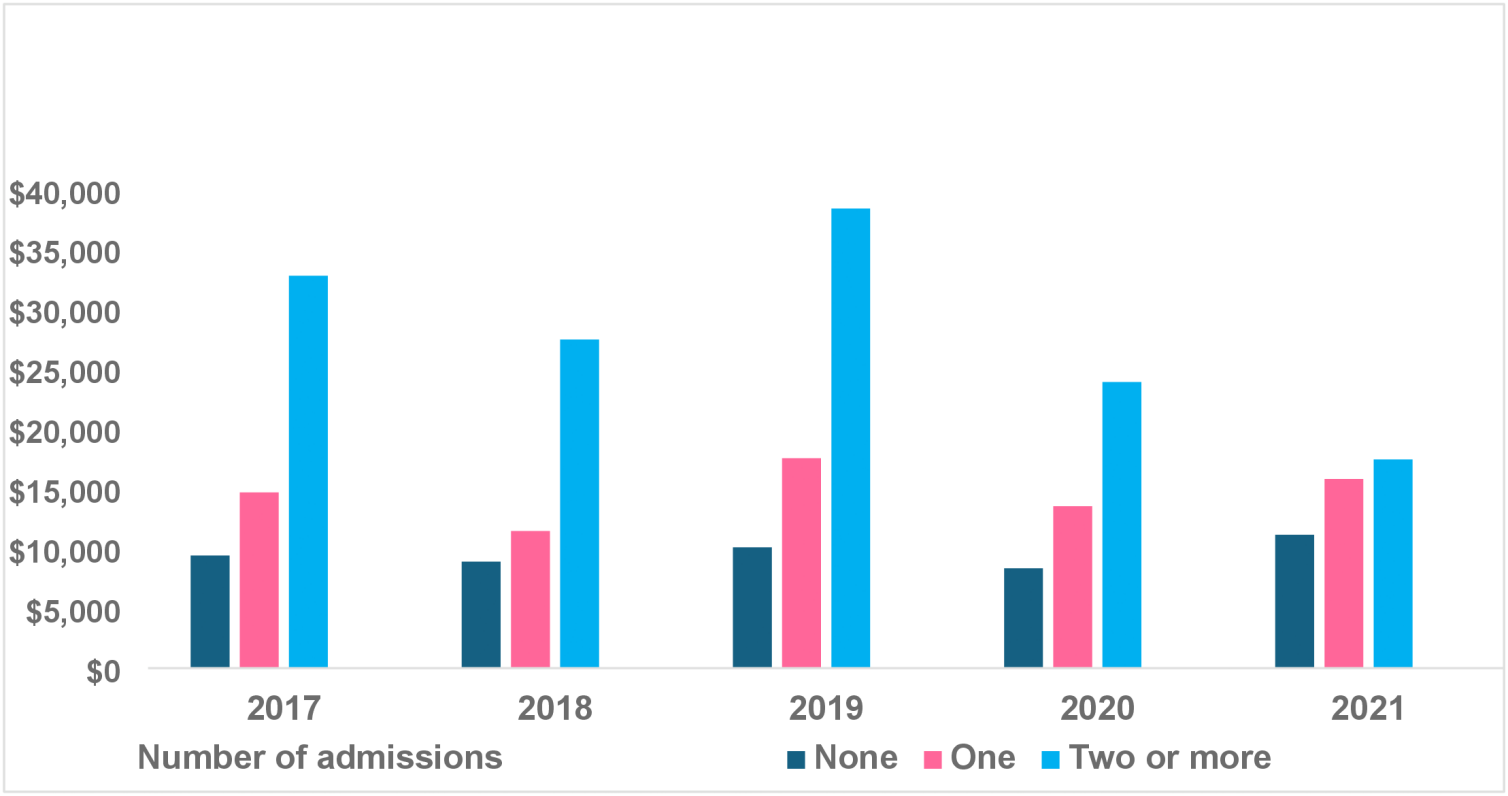
Yearly costs in 2017-2021 for adults according to the number of admissions in 2016.

Fig 13 shows that among individuals admitted in 2016, the average cost for those with one subsequent admission between 2017 and 2021 was $11,400-$15,800, and between $17,400 and $38,400 for those with two or more admissions. In contrast, individuals not admitted in 2016 had average yearly costs of $8,300-$11,000.

Therefore, a comparison of annual healthcare costs between individuals admitted and those not admitted in 2016 indicates that prior-year hospitalization was not associated with significantly higher costs during the subsequent period from 2017 to 2021, after accounting for the number of admissions in those years. These findings indicate that a single admission in 2016 did not independently predict either subsequent admissions or their associated costs.

### Longitudinal analysis of 2016 comorbidity and its impact on hospitalizations and cost

This analysis demonstrates that comorbidity measured in one year can be used to predict both hospitalizations and healthcare costs over the subsequent five years. Comorbidity is a consistent longitudinal predictor of both admissions and cost. In contrast, prior admissions do not reliably predict future admissions. The preceding analyses demonstrate that comorbidity is a significant predictor of both hospital admissions and total healthcare costs.

A mediation analysis was conducted using the framework established by Baron and Kenny [47], to determine whether hospital admissions, a potential mediator, explains part of the association between comorbidity (the independent variable) and total cost, the dependent outcome. Prior analysis shows: 1) that comorbidity significantly predicts the total cost; and 2) comorbidity also significantly predicts hospital admissions from 2017 to 2021. The third condition is that comorbidity (independent) and the admissions (mediator) must significantly predict the total cost (dependent variable) when included in the same regression model. Table 4 does show that the comorbidity score in 2016 and hospital admissions from 2017 to 2021 independently and significantly predict total healthcare costs (all *P* < .01). Therefore, comorbidity in 2016 remains a significant predictor of subsequent healthcare costs; its effect is attenuated when hospital admissions are included in the model, suggesting that admissions partially mediate the relationship.

**Table 4:** Regression analysis assessing criterion (iii) of the Baron and Kenny mediation framework: independent effects of 2016 comorbidity (CCHA 16) and hospital admissions (2017–2021) on the log_10_ transformed total healthcare costs.

	Total cost 2017	Total cost `2018	Total cost 2019	Total cost 2020	Total cost 2021
					
CCHA 2016	.082 ± .006***	.052 ± .007***	.058 ± .006***	.062 ± .007***	.044 ± .007***
					
2017 admission	.879 ± .042***				
					
2018 admission		.557 ± .026***			
					
2019 admission			.487 ± .022***		
					
2020 admission				.502 ± .024***	
					
2021 admission					.558 ± .026***
					
R-squared	0.060	0.132	0.145	0.144	0.130

*** p < 0.01, ** p < 0.05, * p < 0.1

Controlling for age and gender which are significant at P < .01 in each year 2017–2021.

Standard error for regression coefficients are shown.

To further explore this relationship, we employed Generalized Structural Equation Models (GSEMs) to assess both direct and indirect effects over the 2017–2021 period. In the model, comorbidity exerts both a direct effect on total cost and an indirect effect mediated through hospital admissions. The direct effect of comorbidity on cost was statistically significant across all years (all *P* < .01; data in S11 Table. Generalized Structural Equation Model (GSEM) estimates the direct effect of comorbidity on total healthcare costs from 2017 to 2021). Likewise, the indirect effect through hospital admissions was also significant in each year examined (all *P* < .01; data in S12 Table. GSEM estimates of the indirect effect of comorbidity on the log-10 transformed total healthcare costs from 2017 to 2021 mediated by hospital admissions).

Taken together, these findings reinforce the results from the Baron and Kenny approach, indicating that both comorbidity and hospital admissions are significant and consistent predictors of total healthcare costs from 2017 to 2021. In summary, the mediation analysis showed that hospital admissions have a significant role in driving the cost burden associated with comorbidity. Accordingly, interventions aimed at reducing unplanned hospitalizations among individuals with high comorbidity scores may be effective in mitigating downstream healthcare expenditures.

### Comparison of the CCHA to Chronic Conditions Warehouse (CCW30)

**Fig 14:**
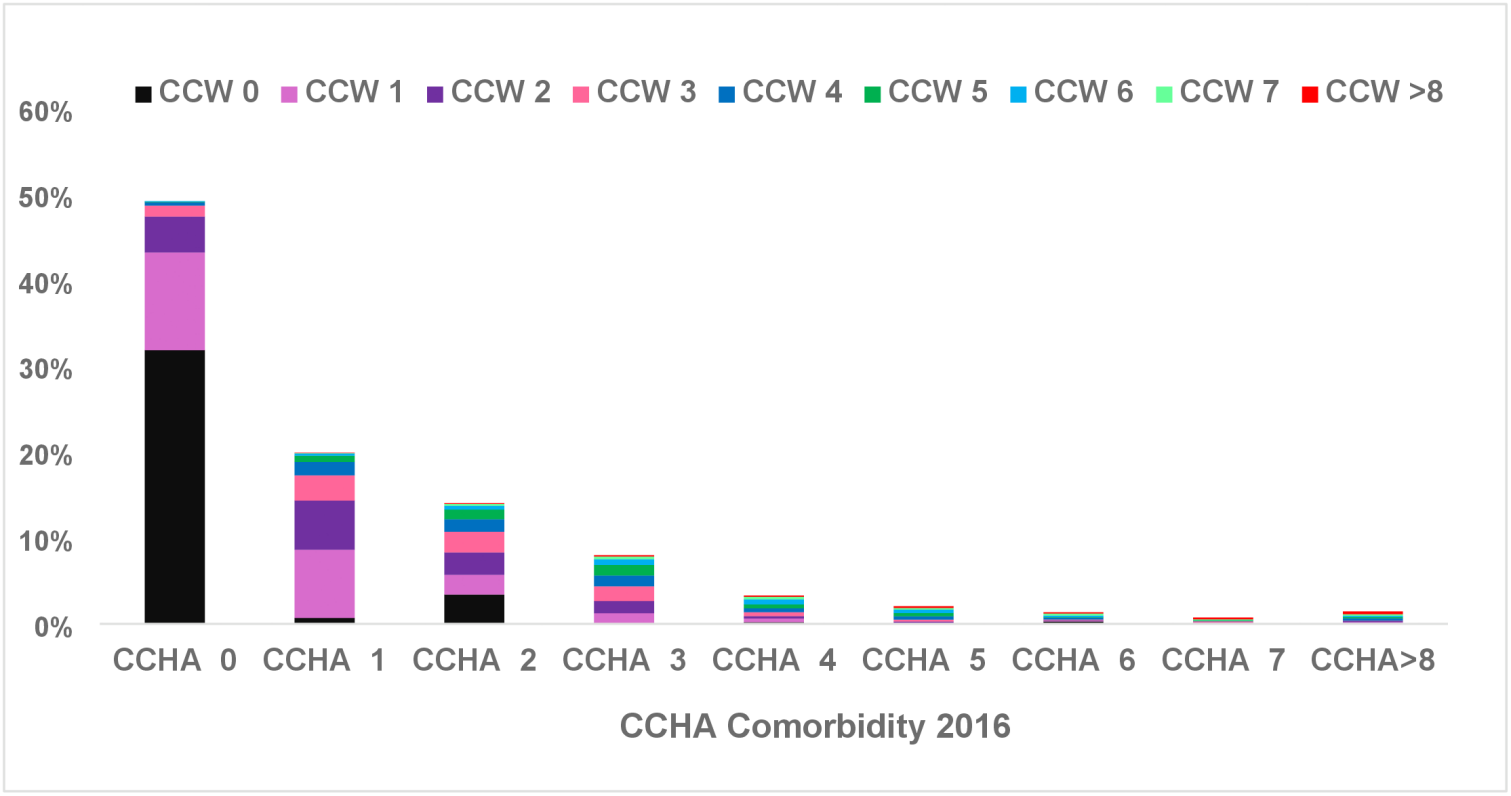
Comparison of 2016 CCHA with 2016 CCW30 2016 Scores: Percent overlap with CCHA.

Fig 14 shows the 2016 CCHA compared to the 2016 Chronic Conditions Warehouse 30 (CCW30). For a CCHA score of zero, there is a range of CCW30 scores, up to CCW30 of ≥8. Although the CCW30 can increase to 30, most of the CCW30 scores are clustered between 0 and 4. Most CCW30 scores are between 0 and 2, although most are scored between 0 and 2 specifically, 36.7% of CCW30 scores are zero; 23.6, one; and 14.6%, two. CCW30 is higher than CCHA in 40.8% of beneficiaries, likely because some of the CCW30 conditions, like cataract and hyperlipidemia, were not drivers of admissions or costs. In contrast, CCHA is higher than CCW30 in 13.8% of beneficiaries.

**Fig 15:**
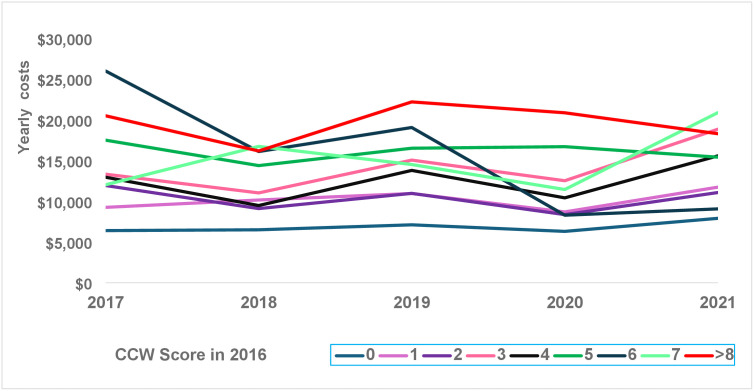
Yearly costs according to the 2016 CCW30 over five years (2017-2021) in the same beneficiaries.

Fig 15 shows the yearly costs according to the CCW30 score in 2016 over the following 5 years (2017–2021). For the most part, the costs were somewhat flat and linear and varied more by year than by CCW30 score. Over the five years, most of the costs were relatively flat, except for the 5.5% with a CCW30 score ≥6 (i.e., 2.7% of 6; 1.8% of 7; and 1.2% of ≥8). In contrast to Fig 15, which shows the costs by CCW30 were relatively flat, Fig 9 shows the yearly costs from 2017 to 2021 increasing steadily (roughly monotonic) according to CCHA.

When the yearly costs from 2017–2021 are graphed by both CCHA and CCW30, the pattern is complex, but the CCW30 did not change the cost pattern within CCHA.

**Fig 16:**
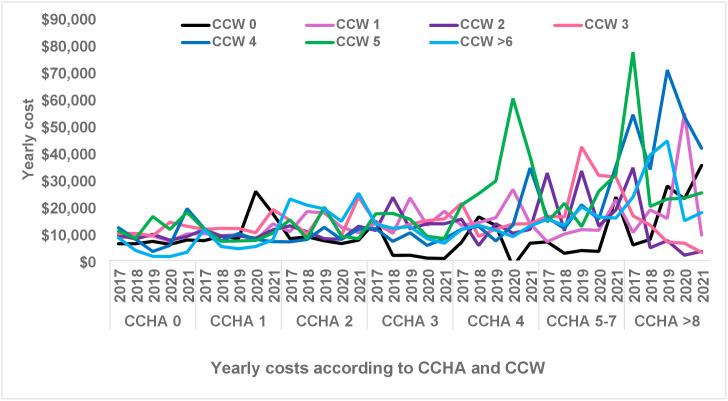
Yearly costs according to 2016 CCHA and 2016 CCW.

In summary, the CCW30 has many conditions that were not cost drivers but increased the total CCW30 score. A comparison of Figs 9 and 15 shows the difference in cost prediction. Fig 15 4G shows that the CCW30 yearly costs by score were fairly flat. Fig 16 shows the side-by-side comparison was extremely scattered, with costs increasingly only with CCHA>5, but CCW30 again flat.

### C.5 Comorbidity from any single year predicts costs, hospitalizations, and repeated hospitalizations in subsequent years

The previous section (C4) demonstrates how comorbidity longitudinally predicts admissions and costs over 6 years among the same cohort of patients. The analysis in this section (C5) takes the first year as beneficiary and then uses the comorbidity from the index year to predict subsequent hospital admission and total cost over the one to five years of follow-up. For clarity, we have called this a *lagged analytic* framework. Adults employed in 2016 could have a maximum five years of follow-up; in 2017, four years; 2018, three years; 2019, two years; and 2020, one year. Data from 2021, the last year of this data, cannot be used, since there is no subsequent year data. This analysis shows how comorbidity from any index year can be employed as a prospective predictor of subsequent yearly costs over variable periods of follow-up. This lagged analysis excludes newborns and obstetrics patients for obvious reasons.

**Fig 17:**
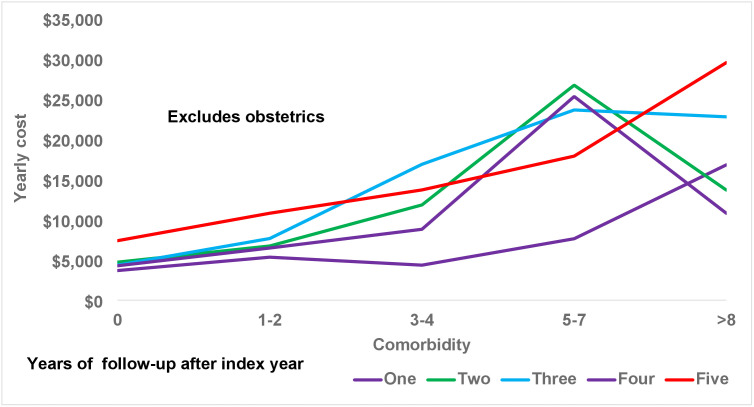
Yearly costs for adults according to comorbidity and the number of follow-ups after the index year.

Table 5 shows the number of adults and children who were beneficiaries according to the total number of years of follow-up after the index year. Fig 17 shows the average yearly cost according to the adults’ index year, comorbidity, and the number of subsequent follow-up years. Average yearly costs rise from $3,600-$7,400 for those with no comorbidity; to $5,300-$10,800 for those with comorbidity scores of 1–2; to $4,400-$13,700 for those with scores of 3–4; to $7,600-$26,000 for those with score of 5–7; and to $13,600-$29,500 with scores ≥8 (P < .01). Yearly costs also rose with more years after the index year. Table 6 shows comorbidity from any specific index year predicting total health care cost in subsequent years according to the lagged years one to five (P < .01 for each lag).

**Table 5:** The framework for the lagged analysis: Number of adults and children according to the total years of follow up after their first or index year as beneficiary.

	Adults	Children	Total
**Index year only**	4,377	1,385	5,762
**Total number of follow-up years after index year**			
One	3,925	1,342	5,627
Two	3,028	1,039	4,067
Three	2,127	753	2,880
Four	1,883	688	2,571
Five	4,860	1,783	6,643
Total	20,200	6,990	27,190

**Table 6:** Comorbidity from the index year predicting log-transformed total healthcare in subsequent (lagged) years: a lagged regression analysis.

	Total cost after 1 year lag	Total cost after 2 year lag	Total cost after 3 year lag	Total cost after 4 year lag	Total cost after 5 year lag
Index year CCHA	.04 ± .01***	.04 ± .02***	.03 ± .02***	.06 ± .02***	.06 ± .01***
					
Observations	3,435	1,204	1,712	1,596	4,483
R-squared	0.03	0.02	0.04	0.05	0.05

***p < 0.01, ** p < 0.05, * p < 0.1

Controlling for age and gender which are significant at P < .01 in each year 2017–2021. Standard error for regression coefficients are shown.

**Fig 18:**
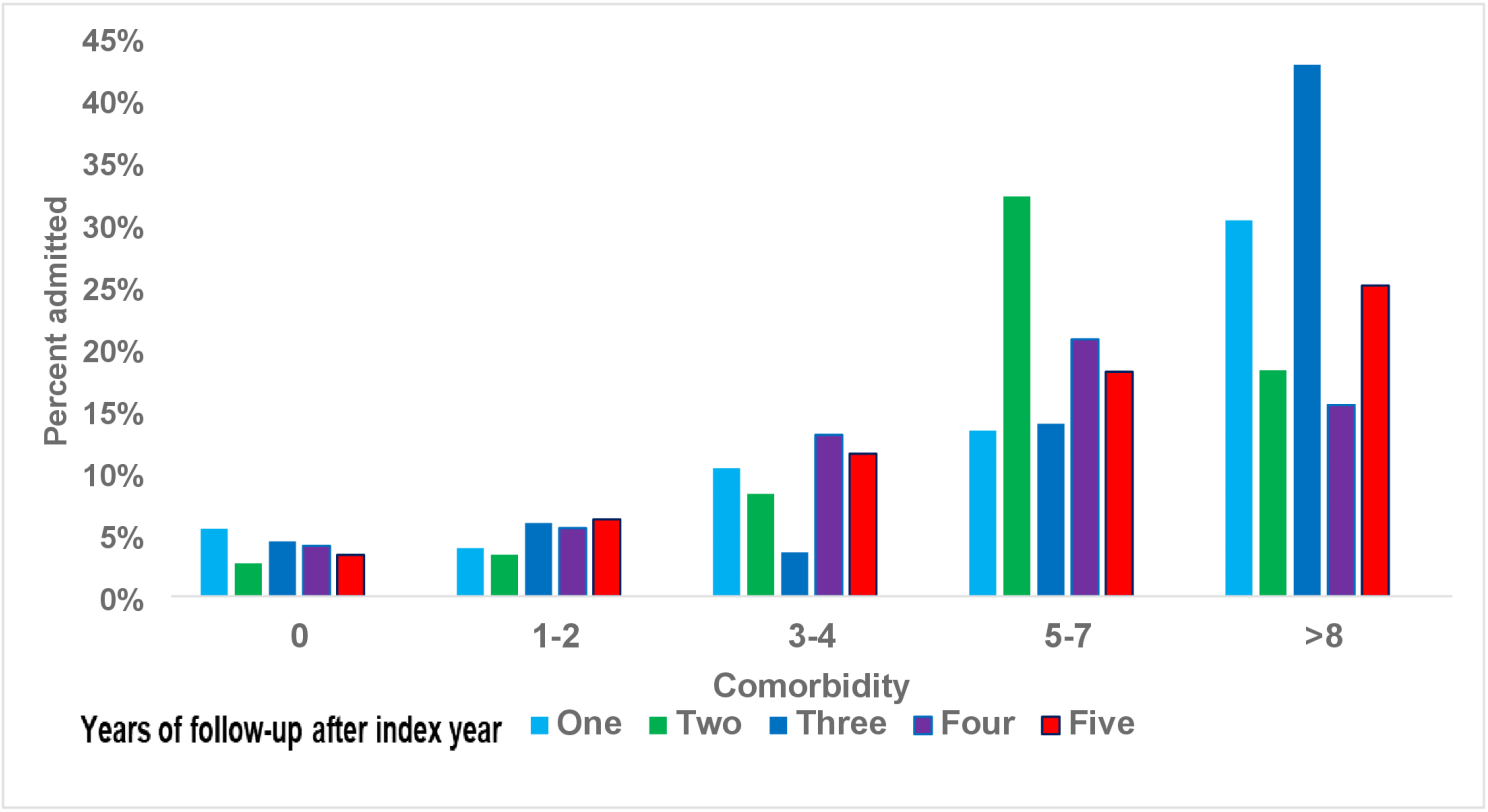
Percent of adults admitted according to comorbidity and the number of years of follow-up, yearly after the index year.

Fig 18 shows the percent of adult beneficiaries admitted according to index year comorbidity and the number of years of follow-up. The percent of adults admitted rose significantly as comorbidity increased from 1%−2% among those with comorbidity of 0; to 2%−4% among those with a score of 1–2; to 7%−9% among those with a score of 3–4; to 10%−20% among those with a score of 5–7; and to 12% to 28% among those with a score of >8 or more. In this lagged analysis, average yearly costs over the years rose from $59,538 in the 5.4% of beneficiaries with one admission to $87,600 in the 1.9% with two admissions, to $118,188 in the 0.8% with three admissions, and to $176,618 in the 1.0% with four or more admissions.

Thus, in a lagged framework, comorbidity from any index year, controlling for age and gender, can be employed as a prospective predictor of subsequent yearly costs (all P < .01; see Table 6) and admissions over the ensuing 1–5 years (all *P* < .01; see Table 7).

**Table 7:** Comorbidity from an index year predicting hospital admissions in subsequent (lagged) years: lagged negative binomial regression analysis.

	Admission after 1-year lag	Admission after 2-year lag	Admission after 3-year lag	Admission after 4-year lag	Admission after 5-year lag
Index year CCHA	.26 ± .05***	.31 ± .07***	.29 ± .06***	.37 ± .07***	.21 ± .03***
					
Observations	3,435	1,204	1,712	1,596	4,483

*** p < 0.01, ** p < 0.05, * p < 0.1

Controlling for age and gender. Age is significant only after a 5 year lag p < .01; gender is significant at p < .01 for1,3,4, and 5 years at p<>01 and at 2 years for P < .05 which are significant at P < .01 in each year 2017–2021.

Standard error for regression coefficients are shown.

## Discussion

This paper focuses on the validation of Charlson Comorbidity Health Analytics, a system and method that utilizes specific weighted definitions of comorbid diseases for adults and children to predict high risk of hospital admissions, repeated admissions, high costs, and often poor outcomes [8]. As shown, the method has validity cross-sectionally in six different years. This is important because serial cross-sections can produce quite different findings when the predictor variable (e.g., comorbidity) differs in its relationship to the outcomes of interest, such as hospital admissions, repeated admissions, and costs. Not all predictor variables have consistent performance over time, but this has practical implications if the findings vary from year to year.

More importantly, the Charlson Comorbidity Health Analytics score from an index year longitudinally predicts the risk of hospitalizations—including repeated hospitalizations—which drive healthcare costs over six years. Therefore, comorbidity from one year can be used to predict admissions and costs longitudinally over six years.

In addition, the lagged analysis demonstrates that comorbidity from any index year can be used to predict subsequent admissions and costs; therefore, it works in dynamic populations, like employers and unions, who have changes in beneficiaries over time.

### Robustness of statistical methods

The statistical methodology integrates the transparent CCHA comorbidity and hospitalization measures that align with empirical distributions of longitudinal utilization and spending. Hospital admissions were analyzed using a zero-inflated negative binomial model, which addresses overdispersion and excess zeros by distinguishing between the probability of structural non-use and admission intensity among those at risk. For total costs, a widely used two-part framework was employed to separate the probability of use from user spending. Among beneficiaries with non-zero expenditures, costs were modeled on the log scale to stabilize variance and reduce sensitivity to extreme values, with the functional form determined by exploratory nonparametric smoothing. Mediation was evaluated within a generalized structural equation modeling framework to estimate both direct (comorbidity→cost) and indirect (comorbidity→admissions→cost) pathways, allowing for appropriate link functions and outcome distributions. Because CCHA is diagnosis-based, and the admission and cost distributions that motivate the zero-inflated negative binomial and two-part models are common across claims settings and time periods, this framework is readily transferable to other populations and suitable for external validation and recalibration.

### Generalizability of the findings

The analyses of diverse populations, including union members, Medicaid, Medicare, and ACO patients, provided the foundational framework for the development of the CCHA and have been published [3–5]. While the CCHA has not been previously published, it has been used collaboratively in several large PCORI-funded studies that support its predictive validity

One of the studies is a PCORI-funded study led by ADVANCE (OCHIN), and OneFlorida had data on more than 2.1 million patients (i.e., 906,000, ADVANCE; 1.2 million, OneFlorida) and was designed to “identify how clinic level measures of comorbidity and social complexity are useful to health system leaders…” and “to assess the association between clinical and social complexity” [49]. The Charlson Comorbidity Health Analytics system was used to evaluate diabetic patients (Table 3, page 604) [50].

The CCHA was also used to evaluate Emergency Department use in Florida and Oregon in 988,106 youth and young adults; those in Oregon had higher CCHA and higher ED use (Table 10) [49]. Adults with a CCHA score ≥8 were 3.5 times more likely to have had an ED visit (Table 11) [49].

The CCHA was also evaluated as a predictor of inpatient admissions and emergency visits in 383,321 adults followed by OCHIN over one year in 2017, as part of the study of comorbidity and social complexity [49]. It was evaluated in 158,559 children and 211,168 adults as a predictor of emergency department visits and hospitalizations [49,50]. In a direct comparison of the Charlson Comorbidity Health Analytics vs. the original Charlson Comorbidity index (which was designed to predict mortality) in 369,737 patients, the Charlson Comorbidity Health Analytics was higher than the original comorbidity index in 37.8% of adults and 17.2% of children [6,7] and the higher differences corresponded with increased admission rates [6,7].

In addition, the CCHA is currently providing the framework for a PCORI-funded cluster randomized trial, “Preventing Tipping Points in high comorbidity patients: A lifeline for health coaches” [8,10] in 1,920 patients from four health systems and four sites per system--16 communities--who have a CCHA score ≥4, in order to prevent unplanned hospitalization and increased disability, comparing a Patient Centered Medical Home (PCMH) to a PCMH with health coaches to help patients achieve life goals and implement self-management strategies [8,10]. In addition, the CCHA has been employed as a framework for primary care panel size management and scheduling at Geisinger [51].

### What insight does it provide? How would it be used in health systems?

Each health system and often each of the practices has different comorbidity distributions and so different insurance providers, such as Medicaid, Medicare, union, employer, commercial, and other insurance. In each of the populations, for many reasons, the distribution of comorbidity varies. Medicare beneficiaries, in general, have higher comorbidity, but it varies from population to population. Not every Medicaid managed care plan, or every Accountable Care Organization, or every network of Community Health Centers has the same comorbidity distribution [3,4].

**Fig 19:**
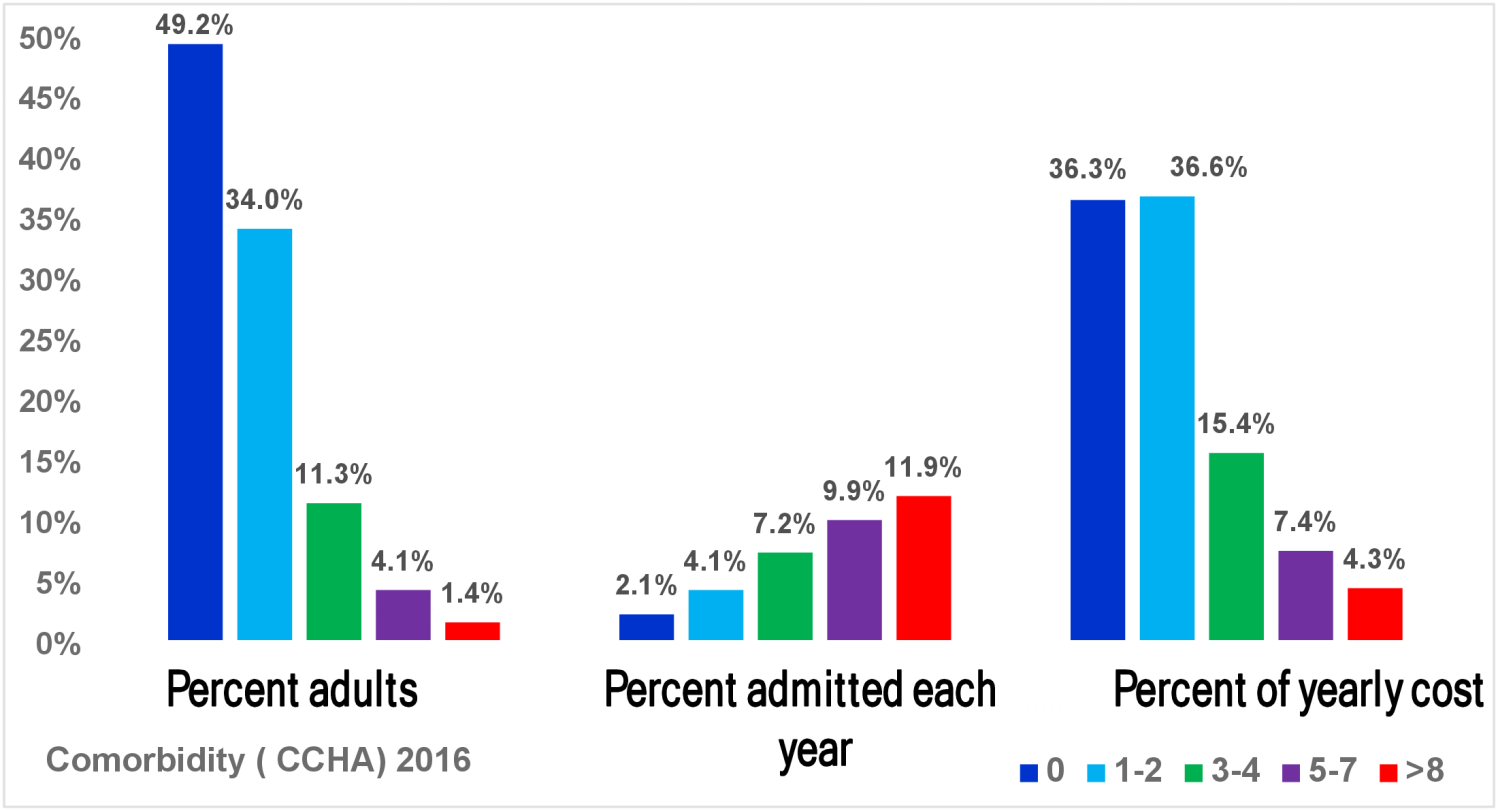
Adult CCHA in 2016: Predicts yearly percent admissions and percent costs over 5 years.

The first step for any population management is to analyze the Comorbidity Health Analytics both in the aggregate in the health systems and then by practice units. The reason is that our previous work suggests that the impact of comorbidity is similar across insurance carriers, but the exact cost curve by comorbidity may vary with a right shift in Medicare, that is, more beneficiaries with higher comorbidity [3–5,7]. However, on an individual level, higher comorbidity is not inevitable at older ages, nor does comorbidity inevitably rise as a person ages. Many ostensible differences in comorbidity over time are due to different look-back periods, especially for ICD-10 claims data. Shown in Fig 19 is the distribution of CCHA in one year (2016) and how CCHA predicts percent yearly admissions and yearly costs over five years. In this population, 5.5% of beneficiaries with CCHA ≥5 had 21.8% of the admissions and 11.7% of the yearly cost.

**Fig 20:**
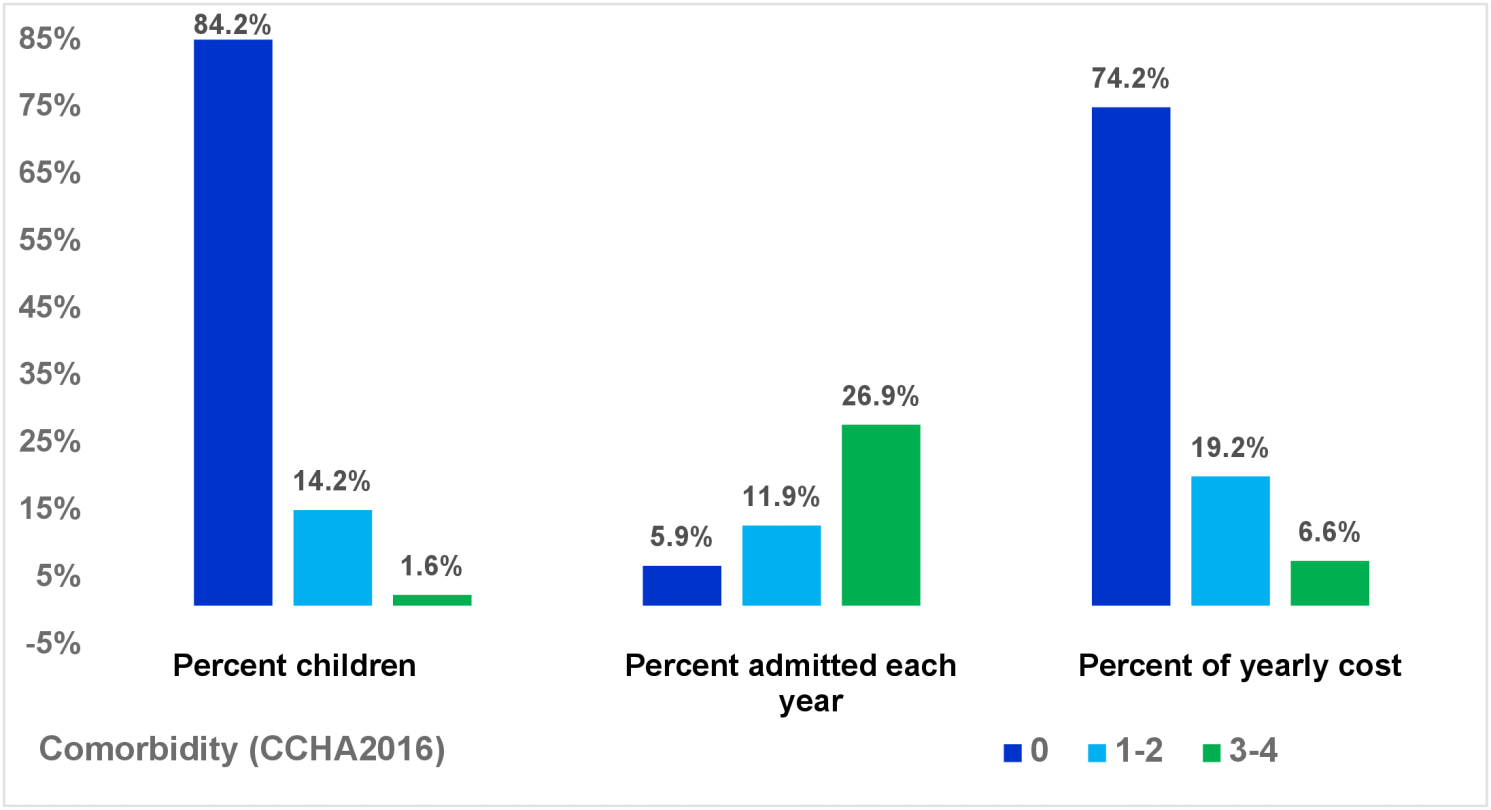
Child CCHA in 2016: Predicts yearly percent admissions and percent costs over 5 years.

Child CCHA is shown in Fig 20, and the shift to the left (i.e., lower scores) is evident in comparison to adults. Among the 1.6% of children with CCHA of 3–4, 26.9% were hospitalized, and they accounted for 6.6% of costs.

Once the CCHA distribution is known in the aggregate, stratified by adults and children (<18 years) and analyzed by provider location, the next step is to identify the conditions driving the highest score. Some of these patients may be receiving or may need specialized treatment for metastatic cancer, transplant, HIV, or severe renal disease (dialysis), and may be cared for in specialized settings, not primary care or family medicine. However, other patients with comorbidity above a population-specific CCHA level, which will vary from population to population, most often require more time and attention from providers. The specific CCHA cut-point (i.e., 4,5,6,7, or ≥8) differs in different populations. Depending on the number of patients in each comorbidity rank, with the goal of providing more intensive care above a threshold number specific to the population, the cut-point has to delineate a manageable number of patients for the specific practice. In adult populations, usually 5%−6% of patients have higher scores with significantly increased admissions and costs.

The CCHA scores do not decrease over time because the included chronic conditions, even if not given ICD-10 codes in a given year, still impact patients’ risk of hospitalization; for example, a diagnosis of breast cancer three years ago, or a myocardial infarction five years ago, continues to impact a patient’s subsequent overall risk.

The fundamental goal is to provide high-comorbidity patients with patient-centered and tailored self-care plans and with relevant self-management education, motivated by the patient’s desire to achieve their own life goals [8]. Patients may also need health coaching and support for life stresses and life events, and for new challenges with social determinants of health [8,10]. It is also important to note that once the population is analyzed and the comorbidity distribution is determined, interventions may vary [8]. The CCHA score is the foundation for population management, which cannot be one size fits all.

Generally, the goal is to prevent destabilization of patients that leads to urgent care or emergency department visits, and then to unplanned hospitalization [8]. However, it is critical to note that there is a difference between planned admissions, such as those for surgery and other procedures, and unplanned admissions. While not every unplanned admission is preventable, it is likely that some unplanned admissions can be prevented by earlier interventions [8,10]. The term “avoidable” admissions is notoriously difficult to define, often reflected in the poor reproducibility of the definition [52]. Clearly, comorbidity is not the only determinant of destabilization. Unmet social determinants of health [10], interval life events [9] and overall frailty [53] can be critical to preventing increase disability and destabilization.

In addition, Comorbidity Health Analytics from an index year can be employed in changing health populations, with new beneficiaries each year and with some beneficiaries leaving the population each year. This analysis replicates how population health management must be grounded to provide a dynamic analysis reflecting population risk changes over time.

### Other strategies to identify patients at high risk

Several different approaches have been used to delineate patients at high risk, including: 1) multiple chronic conditions [41,54–64]; 2) prior hospital admissions, and 3) those with high prior health care costs, especially the upper 5% or 10% of costs. Unfortunately, for the most part, those criteria have not provided the foundation for successful interventions.

Patients with multiple chronic diseases have dramatically increased health care costs as well as increased morbidity in comparison to those without multiple conditions [65–73]. But it’s not all patients with multiple chronic diseases. The other predictive frameworks for identifying high risk populations have focused explicitly on patients with the top 5%−10% of costs or patients with frequent hospitalizations [74–76]. However, as noted by the U.S. Health and Human Services, more than a decade ago, high utilization was not the best predictor of ongoing high risk, because high cost and hospitalization usually decrease in the next year [77]. A Congressional Budget Office analysis also showed that both high prior costs and prior hospitalization overestimate subsequent healthcare costs [78].

### Multiple chronic conditions

Despite agreement that multiple chronic conditions, usually defined as two or more conditions, are a major driver of increased health care utilization, there is no universally accepted definition. An analysis of the MEPS data from 2016–2019 found that 32% of adults have two or more chronic conditions, with 225 common combinations of 20 chronic conditions, and the actual number of combinations is in the millions [79].

Different approaches to defining cogent groups of chronic conditions were reviewed by the Multiple Chronic Disease Working Group in 2010 [54,80]. One of the approaches was the 30 conditions from the Chronic Conditions Warehouse -- CCW30 [41,81]. The Chronic Conditions Warehouse 30 (CCW30) was designed to provide an accessible repository that provided data for research, quality, and policy linked across claims and beneficiary data, including personally identifiable data focused on Medicare and Medicaid patients [82]. Most studies that reported using it focused on specific conditions (i.e., dementia, hypertension, COPD, etc.), not the entire CCW30 spectrum. One study analyzed 710,609 Medicare beneficiaries who were hospitalized at least once between 2005 and 2009 and had two or more chronic conditions, [83] and compared the CCW30, Adjusted Clinical Groups Expanded Diagnostic Clusters, the Medicare Advantage special needs plans (C-SNP), and the Klabunde version of the Charlson Comorbidity Index (which changed the original weights by doubling or tripling for most diseases [13]). Overall, the CCW30 criteria found that 92.3% of beneficiaries met the criteria of ≥2 conditions, while Klabunde CCI, 36.8%; ACG-EDC,18.6%; and c-SNP, 6.2.8% [83]. In a model predicting re-admission within 30 days, the Klabunde CCI and C-SNP had significantly higher odds of 30-day readmission (OR 1.45 and 1.50) compared to the CCW30 (OR 1.10) and ACG (1.22) [83]. In the side-by-side comparison of the CCHA and CCW30, the CCW30 has a broad spectrum of scores across all CCHA ranks. As the CCW30 score increased, costs did not increase significantly and were relatively flat except for the highest CCW30 ranks. When comparing the CCHA with the CCW30 in predicting cost, the CCW30 remains flat. One reason is likely that the CCW30 contains conditions that have a broad spectrum, some of which, such as cataracts or hyperlipidemia, could not reasonably be expected to drive hospitalizations or costs [83].

Other criteria for multiple chronic diseases include the 85 Chronic Condition Indicators [55] and the 70 Hierarchical Condition Codes [38]. These different criteria for multiple chronic conditions have often provided the framework for studies of care coordination and management [77]. CMS-HCC and HHS-HCC provide the basis for reimbursement, either based on the prior year’s ICD codes (CMS-HCC) or the concurrent ICD codes (HHS-HCC). An early Medicare Care Management for High Cost Beneficiaries at MGH focused on patients with multiple hospitalizations per year for an intensive care management program targeting high cost, high complexity patients defined by HCC score with care management, care coordination, and self-management, which showed only small differences in admissions, no impact on re-admission, leading to savings of $288 - $335 a year [27].

For the most part, previous efforts to optimize medical management ostensible targeted to patients with multiple chronic diseases have not reduced hospitalizations or costs. Major case management/care coordination initiatives that have focused on multiple chronic conditions include Chronic Care Clinics, the Chronic Care Model, the Guided Care Model, GRACE, Primary Intensive Management, IMPaCT, and 15 Medicare Coordinated Care Demonstrations [15–32]. Chronic Care Clinics did not have a significant effect on hospitalization [84,85]. The Guided Care did not reduce health care utilization [33,34]. GRACE showed no overall differences in hospitalization [30,31,86]. A VA RCT of a Primary Intensive Management team found no difference between the intensive and the usual care group with regard to inpatient admissions, or ED/inpatient costs [35,87]. The IMPaCT intervention showed no differences in hospitalization [36]. Of the 15 Medicare Coordinated Care Demonstrations [25], only one reduced hospitalization in the initial four years [25–27]. Of the ten Medicare programs with six-year follow-up, only one reduced hospitalization; the other nine did not [28]. There was a modest reduction in admissions in 4 of the 10 programs for certain post-hoc selected high-risk subgroups, but no reduction in cost [25,28]. The interventions included disease management, case management, coordinated care, and other interventions, evaluated over some years, have not had the hoped-for success in achieving either objective [25,33–40]. Of note, Lorig’s Chronic Disease Self-Management, designed to increase patient self-efficacy for managing their own diseases, did reduce hospitalization [88–90].

Most would agree that these initiatives have elements that are important to address the needs of patients with multiple chronic conditions, but they have not had the hoped for successes in patients with multiple chronic diseases. The tremendous heterogeneity in the criteria for defining multiple chronic conditions and the impact of heterogeneity on treatment effects and outcomes clearly impacted all of these studies [91]. It is likely that many of these interventions did not target the relatively small percent of patients at the highest risk [77].

### High admissions or high costs

Two other approaches to identify high-risk patients are to focus on patients with either high admissions or high costs. However, a Congressional Budget Office analysis has shown that these strategies don’t work because both prior costs and prior hospitalizations overestimate subsequent healthcare utilization [78]. They found that among the patients initially in the top 10%, their cost profiles decreased significantly [78]. Others suggest that “segmenting patients based on their baseline clinical risk may be a more fruitful approach rather than segmenting patients once they have become high cost” [92]. An innovative study that combined a cross-sectional and longitudinal analysis of patients who had 3 or more hospitalizations over the prior 12 months or two or more hospitalizations and serious mental illness over 12 months found that at the end of the second year, only 14% remained ‘super-utilizers’ [93]. The difficulty with the focus on ‘super-utilizers’ for analyses of the impact of programs was summarized in an editorial [94]. The problem is that high utilizers in one period often have substantial decreases in health care use subsequently, i.e., “regression to the mean [94].” Study designs that use pre-intervention to post-intervention comparisons are particularly susceptible to this risk of regression to the mean [94,95].

### Limitations

The analysis focuses on 27,190 health care beneficiaries from one institution, who are employees, along with their spouses/partners and children. It does not encompass Medicare, Medicaid, ACO, or other insurers. The comorbidity distribution varies by population and setting, so the exact distributions, admissions, and costs in other populations may differ [3–5,7,8]. While the population distribution, admissions, and costs will vary by insurance (or lack thereof) and setting, the overall predictive pattern remains – the higher the comorbidity, the higher hospital admissions with higher resultant health care costs.

While the sample size in this study is 27,190, increasing the sample size by a multiple of 1,000 to 2.7 million will not normalize the characteristics of the cost curve; that is, it will not reduce the variance. With total costs, there is always a cluster of patients with extremely high costs, and a group with zero or low costs. However, with a much larger sample size, it is much easier to find statistically significant but clinically trivial differences, like a savings of $100 a year. Thus, a larger sample size does not automatically improve external validity.

The claims data analyzed in this study focused on demographics and ICD-10 codes as well as MDC and DRG codes and did not include data extracted from electronic medical records or data on pharmaceutical use, functional, psychosocial, or cognitive status. To prevent confusion, it is important to note that there are at least 16 other quite different versions of the original Charlson index, designed to predict different things, with variable conditions included (I.e., some with or without cardiovascular disease or cancer), and all with different weights and some designed to predict risk for only specific illnesses [13].

### Cross-sectional and Longitudinal analysis

The data from the cross-sectional analysis of data from 2017, 2018, 2019, 2020, and 2021 showed that CCHA comorbidity predicts hospitalizations and costs. It is well established that models often explain a larger portion of variance when based on the same (concurrent) data used for model development, which can lead to overly optimistic conclusions about performance. True evaluation requires examining predictive accuracy on future or unseen data, typically through external validation to obtain an unbiased measure of predictive model performance [96,97].

Importantly, a longitudinal six-year analysis of 2016 beneficiaries showed that comorbidity from 2016 predicted subsequent admissions and higher costs over the entire subsequent five years (2017–2021). Most studies do not evaluate year-to-year differences over five or more years. Moreover, those who were admitted in 2016 did not have persistent higher admission rates over time, nor did those in the top 10% of costs in 2016 have persistent high costs. Only the beneficiaries with higher comorbidity had higher rates of admission and resultant higher costs over five years. In addition, comorbidity from a given year can be used in a lagged manner to predict subsequent admissions and costs; in practice, this is the pattern not only for employers, but also for managed care plans, and also Medicare and Medicaid, as patients move from one plan to another, such as Medicare Advantage to Medicare.

### Summary

A significant burden of comorbidity in adults and children leads to increased admissions and, in turn, high costs [98]. The majority of cost is driven by admissions in adults for non-obstetric care. Comorbidity predicts higher longitudinal costs over six years, but also over any continuous interval of follow-up. Hospital admission is the mediator of cost. Taken together, these findings reinforce the results from the Baron and Kenny approach, indicating that both comorbidity and hospital admissions are significant and consistent predictors of total healthcare costs from 2017 to 2021. Admissions are a mediator of cost; however, although admissions are associated with high resultant cost, it is important to note that a given admission does not predict subsequent admissions or costs.

While admissions for surgery have a higher cost, admissions for medical reasons are twice as common. Medical admissions increase steadily as comorbidity increases above 3 or 4; those with comorbidity levels of 5 or more are often repeatedly admitted. While a few of these patients have specific conditions that place them at vastly higher risk, most have moderate to severe multiple chronic illnesses. In contrast, only a very small proportion of children have increased comorbidity and increased admissions.

The Charlson Comorbidity Health Analytics method provides a prospective method for health systems and population health management programs to identify the specific patients who are at high risk. The standard fifteen-minute primary care visit in patients with high comorbidity, followed by shuffling the patient from specialist to specialist, is often fraught with problems and time after time leads to urgent care visits and unplanned hospitalization. Prevention of destabilization in patients with significant burdens of comorbid disease is possible, but high comorbidity patients require more time and attention [8]. It is important to note that high comorbidity patients may face challenges from social determinants of health [10], life events and stresses [9], as well as frailty [53] which negatively impacts their clinical course. It is important to reemphasize.

Comorbidity drives hospitalization, often repeated hospitalization, and thereby high costs. Simple solutions targeted at one disease have had limited (or no) impact on hospitalizations and costs, and fragmentary, sometimes paradoxical effects on outcomes. Comorbidity is a key determinant of patient prognostic variability, and understanding comorbidity as a driver of performance is critical. High-performance, high-value health care to achieve its overall objectives must be targeted to address the needs of high comorbidity patients, which then holds the potential of reducing hospitalizations and costs.

## Supporting information

S1 Table Predictors of log-transformed total costs in 2016 for adults and children with non-zero expenditures. (DOCX)

S2 Table Predictors for the number of hospital admissions of adult and child admissions in 2016 from zero-inflated negative binomial regression. (DOCX)

S3 Table Predictors of log10 total medical and surgical cross-sectional costs for adults in each year 2017–2021. (DOCX)

S4 Table Predictors of zero total medical and surgical cross-sectional costs for adults for each year 2017–2021. Two-part regression that models zero cost in 2017–2021.
(DOCX)

S5 Table Predictors of log total medical cross-sectional costs for adults in each year 2017–2021. (DOCX)

S6 Table Predictors of zero medical (only) costs for adults cross-sectionally for each year 2017–2021. Two-part regression for zero adult total medical costs (only) in 2017–2021.
(DOCX)

S7 Table Predictors of log total cross-sectional costs for children in each year 2017–2021, eliminating newborns. (DOCX)

S8 Table Predictors of zero cross-sectional costs for children for each year 2017–2021, eliminating newborns. Two-part regression model of zero child total costs that were zero in 2017–2021.
(DOCX)

S9 Table Predictors of adult admissions cross-sectionally 2017–2021, excluding obstetrics. (DOCX)

S10 Table Predictors of child admissions in 2017–2021 cross-sectionally, excluding newborns. (DOCX)

S11 Table Generalized Structural Equation Model (GSEM) estimates the direct effect of comorbidity on total healthcare costs from 2017 to 2021. (DOCX)

S12 Table GSEM estimates of the indirect effect of comorbidity on the log10 transformed total healthcare costs from 2017 to 2021 mediated by hospital admissions. (DOCX)

## References

[1] Feinstein AR. The pre-therapeutic classification of comorbidity in chronic illness. J Chron Dis. 1970;:455–68. 26309916 10.1016/0021-9681(70)90054-8

[2] Charlson ME, Wells MT. Comorbidity: From a Confounder in Longitudinal Clinical Research to the Main Issue in Population Management. Psychother Psychosom. 2022;91(3):145–51. doi: 10.1159/000521952 35196663 PMC9064932

[3] Charlson M, Wells MT, Ullman R, King F, Shmukler C. The Charlson comorbidity index can be used prospectively to identify patients who will incur high future costs. PLoS One. 2014;9(12):e112479. doi: 10.1371/journal.pone.0112479 25469987 PMC4254512

[4] Charlson ME, Wells MT, Kanna B, Dunn V, Michelen W. Medicaid managed care: how to target efforts to reduce costs. BMC Health Serv Res. 2014;14:461. doi: 10.1186/1472-6963-14-461 25395056 PMC4289361

[5] Carrillo JE, Carrillo VA, Guimento R, Mucaria J, Leiman J. The NewYork-Presbyterian Regional Health Collaborative: a three-year progress report. Health Aff (Millwood). 2014;33(11):1985–92. doi: 10.1377/hlthaff.2014.0408 25367994

[6] Cottrell EK, et al. Measuring the effects of social risks on patient health outcomes - a PCORnet(R) study. Measuring the effects of social risks on patient health outcomes - a PCORnet(R) study. Washington, DC. 2021. p. 20–36. 39102490

[7] DeVoe JE, Gold R, Cottrell E, Bauer V, Brickman A, Puro J, et al. The ADVANCE network: accelerating data value across a national community health center network. J Am Med Inform Assoc. 2014;21(4):591–5. doi: 10.1136/amiajnl-2014-002744 24821740 PMC4078289

[8] Charlson ME, Mittleman I, Ramos R, Cassells A, Lin TJ, Eggleston A, et al. Preventing “tipping points” in high comorbidity patients: A lifeline from health coaches - rationale, design and methods. Contemp Clin Trials. 2025;152:107865. doi: 10.1016/j.cct.2025.107865 40024364 PMC12145577

[9] Charlson M, Wells M, Devine CM, Watts J, Ramos R, Hollenberg J, et al. Interval life events are an important determinant of heterogeneity in outcomes in a randomised trial: a novel, simple method of assessment. BMJ Open. 2024;14(7):e074623. doi: 10.1136/bmjopen-2023-074623 39079918 PMC11337663

[10] Charlson ME, Wells MT, Hollenberg J, Ramos R, Martinez GM, Gerard MJ, et al. Examining individual- versus population-level social determinants of health in a cluster randomized trial of health coaches for patients with multiple chronic conditions. J Clin Transl Sci. 2024;8(1):e191. doi: 10.1017/cts.2024.598 39655003 PMC11626581

[11] Charlson ME, Pompei P, Ales KL, MacKenzie CR. A new method of classifying prognostic comorbidity in longitudinal studies: development and validation. J Chronic Dis. 1987;40(5):373–83. doi: 10.1016/0021-9681(87)90171-8 3558716

[12] Van Spall HGC, Toren A, Kiss A, Fowler RA. Eligibility criteria of randomized controlled trials published in high-impact general medical journals: a systematic sampling review. JAMA. 2007;297(11):1233–40. doi: 10.1001/jama.297.11.1233 17374817

[13] Charlson ME, et al. Charlson Comorbidity Index: A Critical Review of Clinimetric Properties. Psychother Psychosom. 2022;91(1):8–35. 34991091 10.1159/000521288

[14] Citation index. https://support.clarivate.com/ScientificandAcademicResearch/s/article/Web-of-Science-Citing-Web-of-Science-data?language=en_US. 2025. Accessed 2025 June 20.

[15] Verdier JM, Byrd V, Stone C. Enhanced primary care case management programs in Medicaid: issues and options for states. 2009.

[16] Brown R. The promise of care coordination models that decrease hospitalizations and improve outcomes of Medicare beneficiaries with chronic illnesses. The National Coalition on Care Coordination. 2009.

[17] Wright K, Hazelett S, Jarjoura D, Allen K. The AD-LIFE trial: working to integrate medical and psychosocial care management models. Home Healthc Nurse. 2007;25(5):308–14. doi: 10.1097/01.NHH.0000269964.34045.d5 17495560

[18] Eng C, Pedulla J, Eleazer GP, McCann R, Fox N. Program of All-inclusive Care for the Elderly (PACE): an innovative model of integrated geriatric care and financing. J Am Geriatr Soc. 1997;45(2):223–32. doi: 10.1111/j.1532-5415.1997.tb04513.x 9033525

[19] Casey PH, Lyle RE, Bird TM, Robbins JM, Kuo DZ, Brown C, et al. Effect of hospital-based comprehensive care clinic on health costs for Medicaid-insured medically complex children. Arch Pediatr Adolesc Med. 2011;165(5):392–8. doi: 10.1001/archpediatrics.2011.5 21300650

[20] Safford MM, Allison JJ, Kiefe CI. Patient complexity: more than comorbidity. the vector model of complexity. J Gen Intern Med. 2007;22 Suppl 3(Suppl 3):382–90. doi: 10.1007/s11606-007-0307-0 18026806 PMC2219701

[21] Sevick MA, Trauth JM, Ling BS, Anderson RT, Piatt GA, Kilbourne AM, et al. Patients with Complex Chronic Diseases: perspectives on supporting self-management. J Gen Intern Med. 2007;22 Suppl 3(Suppl 3):438–44. doi: 10.1007/s11606-007-0316-z 18026814 PMC2150604

[22] Mongan JJ, Ferris TG, Lee TH. Options for slowing the growth of health care costs. N Engl J Med. 2008;358(14):1509–14. doi: 10.1056/NEJMsb0707912 18385503

[23] Bayliss EA, Bosworth HB, Noel PH, Wolff JL, Damush TM, Mciver L. Supporting self-management for patients with complex medical needs: recommendations of a working group. Chronic Illn. 2007;3(2):167–75. doi: 10.1177/1742395307081501 18083671

[24] Sommers LS, Marton KI, Barbaccia JC, Randolph J. Physician, nurse, and social worker collaboration in primary care for chronically ill seniors. Arch Intern Med. 2000;160(12):1825–33. doi: 10.1001/archinte.160.12.1825 10871977

[25] Peikes D, Chen A, Schore J, Brown R. Effects of care coordination on hospitalization, quality of care, and health care expenditures among Medicare beneficiaries: 15 randomized trials. JAMA. 2009;301(6):603–18. doi: 10.1001/jama.2009.126 19211468

[26] Schore J, et al. Fourth report to Congress on the Evaluation of the Medicare Coordinated Care Demonstration. Princeton NJ: Mathematic Policy Research Inc. 2011.

[27] McCall N, Cromwell J, Urato C. Evaluation of care management for high cost beneficiaries (CMHCB) demonstration: Massachusetts General Hospital and Massachusetts General Physicians Organization (MGH) final report. RTI International. 2010.

[28] Brown RS, Peikes D, Peterson G, Schore J, Razafindrakoto CM. Six features of Medicare coordinated care demonstration programs that cut hospital admissions of high-risk patients. Health Aff (Millwood). 2012;31(6):1156–66. doi: 10.1377/hlthaff.2012.0393 22665827

[29] Savitz LA, Bayliss EA. Emerging models of care for individuals with multiple chronic conditions. Health Serv Res. 2021;56 Suppl 1(Suppl 1):980–9. doi: 10.1111/1475-6773.13774 34387358 PMC8515217

[30] Counsell SR, Callahan CM, Buttar AB, Clark DO, Frank KI. Geriatric Resources for Assessment and Care of Elders (GRACE): a new model of primary care for low-income seniors. J Am Geriatr Soc. 2006;54(7):1136–41. doi: 10.1111/j.1532-5415.2006.00791.x 16866688

[31] Counsell SR, Callahan CM, Tu W, Stump TE, Arling GW. Cost analysis of the Geriatric Resources for Assessment and Care of Elders care management intervention. J Am Geriatr Soc. 2009;57(8):1420–6. doi: 10.1111/j.1532-5415.2009.02383.x 19691149 PMC3874584

[32] Peikes D, et al. Third report to Congress on the evaluation of the Medicare coordinated care demonstration. Princeton N.J.: Mathematica Policy Research. 2008.

[33] Boult C, Leff B, Boyd CM, Wolff JL, Marsteller JA, Frick KD, et al. A matched-pair cluster-randomized trial of guided care for high-risk older patients. J Gen Intern Med. 2013;28(5):612–21. doi: 10.1007/s11606-012-2287-y 23307395 PMC3631081

[34] Boult C, Reider L, Frey K, Leff B, Boyd CM, Wolff JL, et al. Early effects of “Guided Care” on the quality of health care for multimorbid older persons: a cluster-randomized controlled trial. J Gerontol A Biol Sci Med Sci. 2008;63(3):321–7. doi: 10.1093/gerona/63.3.321 18375882

[35] Chang ET, Yoon J, Esmaeili A, Zulman DM, Ong MK, Stockdale SE, et al. Outcomes of a randomized quality improvement trial for high-risk Veterans in year two. Health Serv Res. 2021;56 Suppl 1(Suppl 1):1045–56. doi: 10.1111/1475-6773.13674 34145564 PMC8515223

[36] Kangovi S, Mitra N, Norton L, Harte R, Zhao X, Carter T, et al. Effect of Community Health Worker Support on Clinical Outcomes of Low-Income Patients Across Primary Care Facilities: A Randomized Clinical Trial. JAMA Intern Med. 2018;178(12):1635–43. doi: 10.1001/jamainternmed.2018.4630 30422224 PMC6469661

[37] Vasan A, Morgan JW, Mitra N, Xu C, Long JA, Asch DA, et al. Effects of a standardized community health worker intervention on hospitalization among disadvantaged patients with multiple chronic conditions: A pooled analysis of three clinical trials. Health Serv Res. 2020;55 Suppl 2(Suppl 2):894–901. doi: 10.1111/1475-6773.13321 32643163 PMC7518822

[38] Pope GC, Kautter J, Ellis RP, Ash AS, Ayanian JZ, Lezzoni LI, et al. Risk adjustment of Medicare capitation payments using the CMS-HCC model. Health Care Financ Rev. 2004;25(4):119–41. 15493448 PMC4194896

[39] Berkowitz SA, Parashuram S, Rowan K, Andon L, Bass EB, Bellantoni M, et al. Association of a Care Coordination Model With Health Care Costs and Utilization: The Johns Hopkins Community Health Partnership (J-CHiP). JAMA Netw Open. 2018;1(7):e184273. doi: 10.1001/jamanetworkopen.2018.4273 30646347 PMC6324376

[40] Wang L, Porter B, Maynard C, Evans G, Bryson C, Sun H, et al. Predicting risk of hospitalization or death among patients receiving primary care in the Veterans Health Administration. Med Care. 2013;51(4):368–73. doi: 10.1097/MLR.0b013e31827da95a 23269113

[41] C. C. C. Chronic Condition Warehouses. https://www2.ccwdata.org/web/guest/home/. 2017. Accessed 2025.

[42] Medicaid CMA. Chronic Conditions Administrative Data User Guide Version 4.0. 2025. chr-chronic-condition-algorithms-1.pdf

[43] Communication P. 2019.

[44] Long DL, Preisser JS, Herring AH, Golin CE. A marginalized zero-inflated Poisson regression model with overall exposure effects. Stat Med. 2014;33(29):5151–65. doi: 10.1002/sim.6293 25220537 PMC4227945

[45] Mihaylova B, Briggs A, O’Hagan A, Thompson SG. Review of statistical methods for analysing healthcare resources and costs. Health Econ. 2011;20(8):897–916. doi: 10.1002/hec.1653 20799344 PMC3470917

[46] Cragg JG. Some Statistical Models for Limited Dependent Variables with Application to the Demand for Durable Goods. Econometrica. 1971;39(5):829. doi: 10.2307/1909582

[47] Baron RM, Kenny DA. The moderator-mediator variable distinction in social psychological research: conceptual, strategic, and statistical considerations. J Pers Soc Psychol. 1986;51(6):1173–82. doi: 10.1037//0022-3514.51.6.1173 3806354

[48] Rabe-Hesketh S, Skrondal A. Multilevel and Longitudinal Modeling Using Stata. 3rd Edition ed. Stata Press. 2012.

[49] Cottrell EK, et al. Measuring the effects of social risks on patient health outcomes - a PCORnet(R) study. Washington, DC. 2021. 39102490

[50] Cottrell EK, et al. The impact of social and clinical complexity on diabetes control measures. J Am Board Fam Med. 2020;33(4):600–10. 32675271 10.3122/jabfm.2020.04.190367

[51] Johannes B, Pacheco C, Hawthorne S, Kobylinski M, Strevig G, Kubasti T, et al. Charlson Comorbidity Health Analytics (CCHA) and Needs-Based Segmentation (NBS): A Strategy for Managing Primary Care Panel Complexity. Ann Fam Med. 2026;24(2):169. doi: 10.1370/afm.250420 41876102 PMC13008794

[52] Clubbs Coldron B, MacRury S, Coates V, Khamis A. Redefining avoidable and inappropriate admissions. Public Health. 2022;202:66–73. doi: 10.1016/j.puhe.2021.11.004 34906791

[53] Fried LP, Ferrucci L, Darer J, Williamson JD, Anderson G. Untangling the concepts of disability, frailty, and comorbidity: implications for improved targeting and care. J Gerontol A Biol Sci Med Sci. 2004;59(3):255–63. doi: 10.1093/gerona/59.3.m255 15031310

[54] Goodman RA, Posner SF, Huang ES, Parekh AK, Koh HK. Defining and measuring chronic conditions: imperatives for research, policy, program, and practice. Prev Chronic Dis. 2013;10:E66. doi: 10.5888/pcd10.120239 23618546 PMC3652713

[55] Hwang W, Weller W, Ireys H, Anderson G. Out-of-pocket medical spending for care of chronic conditions. Health Aff (Millwood). 2001;20(6):267–78. doi: 10.1377/hlthaff.20.6.267 11816667

[56] Egan BM, Sutherland SE, Tilkemeier PL, Davis RA, Rutledge V, Sinopoli A. A cluster-based approach for integrating clinical management of Medicare beneficiaries with multiple chronic conditions. PLoS One. 2019;14(6):e0217696. doi: 10.1371/journal.pone.0217696 31216301 PMC6584004

[57] Ho IS-S, Azcoaga-Lorenzo A, Akbari A, Black C, Davies J, Hodgins P, et al. Examining variation in the measurement of multimorbidity in research: a systematic review of 566 studies. Lancet Public Health. 2021;6(8):e587–97. doi: 10.1016/S2468-2667(21)00107-9 34166630

[58] Violan C, Foguet-Boreu Q, Flores-Mateo G, Salisbury C, Blom J, Freitag M, et al. Prevalence, determinants and patterns of multimorbidity in primary care: a systematic review of observational studies. PLoS One. 2014;9(7):e102149. doi: 10.1371/journal.pone.0102149 25048354 PMC4105594

[59] Johnston MC, Crilly M, Black C, Prescott GJ, Mercer SW. Defining and measuring multimorbidity: a systematic review of systematic reviews. Eur J Public Health. 2019;29(1):182–9. doi: 10.1093/eurpub/cky098 29878097

[60] Arnett DK, et al. AHA/ACC/HHS strategies to enhance application of clinical practice guidelines in patients with cardiovascular disease and comorbid conditions: from the American Heart Association, American College of Cardiology, and U.S. Department of Health and Human Services. J Am Coll Cardiol. 2014;64(17):1851–6. 25219921 10.1016/j.jacc.2014.07.012

[61] Skou ST, et al. Multimorbidity. Nat Rev Dis Primers. 2022;8(1):48. 35835758 10.1038/s41572-022-00376-4 PMC7613517

[62] Forman DE, Maurer MS, Boyd C, Brindis R, Salive ME, Horne FM, et al. Multimorbidity in Older Adults With Cardiovascular Disease. J Am Coll Cardiol. 2018;71(19):2149–61. doi: 10.1016/j.jacc.2018.03.022 29747836 PMC6028235

[63] Stirland LE, González-Saavedra L, Mullin DS, Ritchie CW, Muniz-Terrera G, Russ TC. Measuring multimorbidity beyond counting diseases: systematic review of community and population studies and guide to index choice. BMJ. 2020;368:m160. doi: 10.1136/bmj.m160 32071114 PMC7190061

[64] Boyd CM, Fortin M. Future of multimorbidity research: how should understanding of multimorbidity inform health system design. Public Health Review. 2010;32:451–74.

[65] Marengoni A, Angleman S, Melis R, Mangialasche F, Karp A, Garmen A, et al. Aging with multimorbidity: a systematic review of the literature. Ageing Res Rev. 2011;10(4):430–9. doi: 10.1016/j.arr.2011.03.003 21402176

[66] Glynn LG, Buckley B, Reddan D, Newell J, Hinde J, Dinneen SF, et al. Multimorbidity and risk among patients with established cardiovascular disease: a cohort study. Br J Gen Pract. 2008;58(552):488–94. doi: 10.3399/bjgp08X319459 18611315 PMC2441510

[67] Fillenbaum GG, Pieper CF, Cohen HJ, Cornoni-Huntley JC, Guralnik JM. Comorbidity of five chronic health conditions in elderly community residents: determinants and impact on mortality. J Gerontol A Biol Sci Med Sci. 2000;55(2):M84-9. doi: 10.1093/gerona/55.2.m84 10737690

[68] Gijsen R, Hoeymans N, Schellevis FG, Ruwaard D, Satariano WA, van den Bos GA. Causes and consequences of comorbidity: a review. J Clin Epidemiol. 2001;54(7):661–74. doi: 10.1016/s0895-4356(00)00363-2 11438406

[69] Menotti A, Mulder I, Nissinen A, Giampaoli S, Feskens EJ, Kromhout D. Prevalence of morbidity and multimorbidity in elderly male populations and their impact on 10-year all-cause mortality: The FINE study (Finland, Italy, Netherlands, Elderly). J Clin Epidemiol. 2001;54(7):680–6. doi: 10.1016/s0895-4356(00)00368-1 11438408

[70] Majumdar UB, Hunt C, Doupe P, Baum AJ, Heller DJ, Levine EL, et al. Multiple chronic conditions at a major urban health system: a retrospective cross-sectional analysis of frequencies, costs and comorbidity patterns. BMJ Open. 2019;9(10):e029340. doi: 10.1136/bmjopen-2019-029340 31619421 PMC6797368

[71] Wallace RB, Salive ME. The dimensions of multiple chronic conditions: where do we go from here? A commentary on the Special Issue of Preventing Chronic Disease. Prev Chronic Dis. 2013;10:E59. doi: 10.5888/pcd10.130104 23618539 PMC3652714

[72] Schneider KM, O’Donnell BE, Dean D. Prevalence of multiple chronic conditions in the United States’ Medicare population. Health Qual Life Outcomes. 2009;7:82. doi: 10.1186/1477-7525-7-82 19737412 PMC2748070

[73] Grembowski D, Schaefer J, Johnson KE, Fischer H, Moore SL, Tai-Seale M, et al. A conceptual model of the role of complexity in the care of patients with multiple chronic conditions. Med Care. 2014;52 Suppl 3:S7–14. doi: 10.1097/MLR.0000000000000045 24561762

[74] Wammes JJG, van der Wees PJ, Tanke MAC, Westert GP, Jeurissen PPT. Systematic review of high-cost patients’ characteristics and healthcare utilisation. BMJ Open. 2018;8(9):e023113. doi: 10.1136/bmjopen-2018-023113 30196269 PMC6129088

[75] Berkman ND, et al. Management of high-need, high-cost patients: A “best fit” framework synthesis, realist review, and systematic reviews. AHRQ. 2021. 34780127

[76] Hong C, Siegel A, Ferris T. *Caring for High-need, High-cost Patients: What Makes for a Successful Care Management Program?* Issue Brief (Commonwealth Fund). 2014;19: pp. 1–19. 25115035

[77] Framework HMS. Identifying and Stratifying Individuals with Multiple Chronic Conditions for Care Management. Washington, DC: Department of Health and Human Services. 2012.

[78] Lee J, Anderson T. High Cost Medicare Beneficiaries. Washington DC: Congressional Budget Office. 2005.

[79] Schiltz NK. Prevalence of multimorbidity combinations and their association with medical costs and poor health: A population-based study of U.S. adults. Front Public Health. 2022;10:953886. doi: 10.3389/fpubh.2022.953886 36466476 PMC9717681

[80] Multiple chronic conditions: a strategic framework. U.S. Department of Health and Human Services. 2010. https://www.hhs.gov/sites/default/files/ash/initiatives/mcc/mcc_framework.pdf

[81] CMS.gov. Multiple chronic conditions measures. https://www.cms.gov. 2023.

[82] CMS P. PIA for Chronic Conditions Warehouse. https://www.ccwdata.org. 2024.

[83] Dattalo M, DuGoff E, Ronk K, Kennelty K, Gilmore-Bykovskyi A, Kind AJ. Apples and Oranges: Four Definitions of Multiple Chronic Conditions and their Relationship to 30-Day Hospital Readmission. J Am Geriatr Soc. 2017;65(4):712–20. doi: 10.1111/jgs.14539 28205206 PMC5397355

[84] Coleman EA, Grothaus LC, Sandhu N, Wagner EH. Chronic care clinics: a randomized controlled trial of a new model of primary care for frail older adults. J Am Geriatr Soc. 1999;47(7):775–83. doi: 10.1111/j.1532-5415.1999.tb03832.x 10404919

[85] Coleman EA, Eilertsen TB, Kramer AM, Magid DJ, Beck A, Conner D. Reducing emergency visits in older adults with chronic illness. A randomized, controlled trial of group visits. Eff Clin Pract. 2001;4(2):49–57. 11329985

[86] Counsell SR, Callahan CM, Clark DO, Tu W, Buttar AB, Stump TE, et al. Geriatric care management for low-income seniors: a randomized controlled trial. JAMA. 2007;298(22):2623–33. doi: 10.1001/jama.298.22.2623 18073358

[87] Zulman DM, Chang ET, Wong A, Yoon J, Stockdale SE, Ong MK, et al. Effects of Intensive Primary Care on High-Need Patient Experiences: Survey Findings from a Veterans Affairs Randomized Quality Improvement Trial. J Gen Intern Med. 2019;34(Suppl 1):75–81. doi: 10.1007/s11606-019-04965-0 31098977 PMC6542922

[88] Lorig KR, Ritter P, Stewart AL, Sobel DS, Brown BW Jr, Bandura A, et al. Chronic disease self-management program: 2-year health status and health care utilization outcomes. Med Care. 2001;39(11):1217–23. doi: 10.1097/00005650-200111000-00008 11606875

[89] Lorig KR, Sobel DS, Stewart AL, Brown BW Jr, Bandura A, Ritter P, et al. Evidence suggesting that a chronic disease self-management program can improve health status while reducing hospitalization: a randomized trial. Med Care. 1999;37(1):5–14. doi: 10.1097/00005650-199901000-00003 10413387

[90] Lorig KR, Sobel DS, Ritter PL, Laurent D, Hobbs M. Effect of a self-management program on patients with chronic disease. Eff Clin Pract. 2001;4(6):256–62. 11769298

[91] Maciejewski ML, Bayliss EA. Approaches to comparative effectiveness research in multimorbid populations. Med Care. 2014;52 Suppl 3:S23-30. doi: 10.1097/MLR.0000000000000060 24561754

[92] Joynt KE, Figueroa JF, Beaulieu N, Wild RC, Orav EJ, Jha AK. Segmenting high-cost Medicare patients into potentially actionable cohorts. Healthc (Amst). 2017;5(1–2):62–7. doi: 10.1016/j.hjdsi.2016.11.002 27914968

[93] Johnson TL, Rinehart DJ, Durfee J, Brewer D, Batal H, Blum J, et al. For many patients who use large amounts of health care services, the need is intense yet temporary. Health Aff (Millwood). 2015;34(8):1312–9. doi: 10.1377/hlthaff.2014.1186 26240244

[94] Katz MH. Trust but Verify (Ideally With a Randomized Clinical Trial). JAMA Intern Med. 2017;177(2):162–3. doi: 10.1001/jamainternmed.2016.8433 28027342

[95] Horn BP, Crandall C, Moffett M, Hensley M, Howarth S, Binder DS, et al. The Economic Impact of Intensive Care Management for High-Cost Medically Complex Patients: An Evaluation of New Mexico’s Care One Program. Popul Health Manag. 2016;19(6):398–404. doi: 10.1089/pop.2015.0142 27031738

[96] Hastie T, Tibshirani R, Friedman J. The elements of statistical learning: Data mining, inference, and prediction. Springer. 2009.

[97] Harrell FE. Regression Modeling Strategies. Springer. 2015.

[98] Charlson M, Charlson RE, Briggs W, Hollenberg J. Can disease management target patients most likely to generate high costs? The impact of comorbidity. J Gen Intern Med. 2007;22(4):464–9. doi: 10.1007/s11606-007-0130-7 17372794 PMC1829434

