# Supplementary material for: Charlson comorbidity health analytics: A population management strategy to identify risk of hospitalizations, repeated hospitalizations, and resultant high cost
Source: PLoS One. 2026 Jun 29;21(6):e0351956. doi: 10.1371/journal.pone.0351956 (PMC13313358; doi:10.1371/journal.pone.0351956)
Supplement: S1 Table — (DOCX) [file pone.0351956.s001.docx]

**S1 Table. Predictors of log_10_-transformed total costs in 2016 for adults and children with non-zero expenditures.**

|  |  |  |
| --- | --- | --- |
|  | **Adult total 2016 cost** | **Child total 2016 cost** |
| CCHA16 | -.048+-.002*** | -.034+-.016*** |
|  |  |  |
| Observations | 9,823 | 3,307 |
| R-squared | .084 | .123 |
|  |  |  |
| *** p<0.01, ** p<0.05, * p<0.1  Controlling for age and gender. Age p<.01; gender is significant at p<.01 only for adults | | |
