# Supplementary material for: Charlson comorbidity health analytics: A population management strategy to identify risk of hospitalizations, repeated hospitalizations, and resultant high cost
Source: PLoS One. 2026 Jun 29;21(6):e0351956. doi: 10.1371/journal.pone.0351956 (PMC13313358; doi:10.1371/journal.pone.0351956)
Supplement: S2 Table — (DOCX) [file pone.0351956.s002.docx]

**S2 Table. Predictors for the number of hospital admissions of adult and child admissions in 2016 from zero-inflated negative binomial regression.**

|  |  | |  | |  | |
| --- | --- | --- | --- | --- | --- | --- |
|  |  | |  | |  | |
|  |  | | **Adult Admissions** | | **Child Admissions** | |
|  | CCHA2016 | | .304+.235*** | | .479+.201*** | |
|  |  | |  | |  | |
|  | Observations | | 7,471 | | 2,583 | |
|  |  | |  | |  | |
| Controlling for age and gender, age p<.01 | |  | |  | |  |
| Standard errors i*** p<0.01, ** p<0.05, * p<0.1 | | | | | |  |
