# Supplementary material for: Charlson comorbidity health analytics: A population management strategy to identify risk of hospitalizations, repeated hospitalizations, and resultant high cost
Source: PLoS One. 2026 Jun 29;21(6):e0351956. doi: 10.1371/journal.pone.0351956 (PMC13313358; doi:10.1371/journal.pone.0351956)
Supplement: S3 Table — (DOCX) [file pone.0351956.s003.docx]

**S3 Table. Predictors of log_10_ total medical and surgical cross-sectional costs for adults in each year 2017-2021**.

|  |  | **Medical and surgical** | **Medical and surgical** | **Medical and surgical** | **Medical and surgical** | **Medical and surgical** |
| --- | --- | --- | --- | --- | --- | --- |
|  |  | **Cost 2017** | **Cost 2018** | **Cost 2019** | **Cost 2020** | **Cost 2021** |
|  |  |  |  |  |  |  |
|  | CCHA2017 | .347+-.011*** |  |  |  |  |
|  |  |  |  |  |  |  |
|  | CCHA2018 |  | .353+-.012*** |  |  |  |
|  |  |  |  |  |  |  |
|  | CCHA2019 |  |  | .368+-.011*** |  |  |
|  |  |  |  |  |  |  |
|  | CCHA2020 |  |  |  | .414+-.011*** |  |
|  |  |  |  |  |  |  |
|  | CCHA2021 |  |  |  |  | .432+-.010*** |
|  |  |  |  |  |  |  |
|  | Observations | 10,331 | 10,604 | 11,042 | 11485 | 12735 |
|  | R-squared | .114 | .097 | .116 | .128 | .142 |
|  |  |  |  |  |  |  |
|  | *** p<0.01, ** p<0.05, * p<0.1 | | |  |  |  |

Controlling for age and gender, age p<.01 for 2017-2021; gender p<01 for 2017-2020
