# Supplementary material for: Charlson comorbidity health analytics: A population management strategy to identify risk of hospitalizations, repeated hospitalizations, and resultant high cost
Source: PLoS One. 2026 Jun 29;21(6):e0351956. doi: 10.1371/journal.pone.0351956 (PMC13313358; doi:10.1371/journal.pone.0351956)
Supplement: S5 Table — (DOCX) [file pone.0351956.s005.docx]

**S5 Table. Predictors of log_10_ total medical cross-sectional costs for adults in each year 2017-2021.**

|  |  |  |  |  |  |  |
| --- | --- | --- | --- | --- | --- | --- |
|  |  | **Total medical** | **Total medical** | **Total medical** | **Total medical** | **Total medical** |
|  |  | **Cost 2017** | **Cost 2018** | **Cost 2019** | **Cost** | **Cost 2021** |
|  |  |  |  |  |  |  |
|  | CCHA2017 | .297+-.011*** |  |  |  |  |
|  |  |  |  |  |  |  |
|  | CCHA2018 |  | .299+-.012*** |  |  |  |
|  |  |  |  |  |  |  |
|  | CCHA2019 |  |  | .308+-.011*** |  |  |
|  |  |  |  |  |  |  |
|  | CCHA2020 |  |  |  | .364+-.012*** |  |
|  |  |  |  |  |  |  |
|  | CCHA2021 |  |  |  |  | .383+-.011*** |
|  |  |  |  |  |  |  |
|  |  |  |  |  |  |  |
|  | Observations | 10,128 | 10,412 | 10,832 | 11,326 | 12,535 |
|  | R-squared | .111 | .101 | .108 | .124 | .133 |
|  |  |  |  |  |  |  |
|  | *** p<0.01, ** p<0.05, * p<0.1Controlling for age and gender, age p<.01 for 2017-2021; gender <01 for 2017-2021for 2017-2021 | | |  |  |  |
