# Supplementary material for: Charlson comorbidity health analytics: A population management strategy to identify risk of hospitalizations, repeated hospitalizations, and resultant high cost
Source: PLoS One. 2026 Jun 29;21(6):e0351956. doi: 10.1371/journal.pone.0351956 (PMC13313358; doi:10.1371/journal.pone.0351956)
Supplement: S6 Table — Two-part regression for zero adult total medical costs (only) in 2017–2021. (DOCX) [file pone.0351956.s006.docx]

**Supporting Information**

**S1 Table. Predictors of log_10_-transformed total costs in 2016 for adults and children with non-zero expenditures.**

|  |  |  |
| --- | --- | --- |
|  | **Adult total 2016 cost** | **Child total 2016 cost** |
| CCHA16 | -.048+-.002*** | -.034+-.016*** |
|  |  |  |
| Observations | 9,823 | 3,307 |
| R-squared | .084 | .123 |
|  |  |  |
| *** p<0.01, ** p<0.05, * p<0.1  Controlling for age and gender. Age p<.01; gender is significant at p<.01 only for adults | | |

**S2 Table. Predictors for the number of hospital admissions of adult and child admissions in 2016 from zero-inflated negative binomial regression.**

|  |  | |  | |  | |
| --- | --- | --- | --- | --- | --- | --- |
|  |  | |  | |  | |
|  |  | | **Adult Admissions** | | **Child Admissions** | |
|  | CCHA2016 | | .304+.235*** | | .479+.201*** | |
|  |  | |  | |  | |
|  | Observations | | 7,471 | | 2,583 | |
|  |  | |  | |  | |
| Controlling for age and gender, age p<.01 | |  | |  | |  |
| Standard errors i*** p<0.01, ** p<0.05, * p<0.1 | | | | | |  |

**S3 Table. Predictors of log_10_ total medical and surgical cross-sectional costs for adults in each year 2017-2021**.

|  |  | **Medical and surgical** | **Medical and surgical** | **Medical and surgical** | **Medical and surgical** | **Medical and surgical** |
| --- | --- | --- | --- | --- | --- | --- |
|  |  | **Cost 2017** | **Cost 2018** | **Cost 2019** | **Cost 2020** | **Cost 2021** |
|  |  |  |  |  |  |  |
|  | CCHA2017 | .347+-.011*** |  |  |  |  |
|  |  |  |  |  |  |  |
|  | CCHA2018 |  | .353+-.012*** |  |  |  |
|  |  |  |  |  |  |  |
|  | CCHA2019 |  |  | .368+-.011*** |  |  |
|  |  |  |  |  |  |  |
|  | CCHA2020 |  |  |  | .414+-.011*** |  |
|  |  |  |  |  |  |  |
|  | CCHA2021 |  |  |  |  | .432+-.010*** |
|  |  |  |  |  |  |  |
|  | Observations | 10,331 | 10,604 | 11,042 | 11485 | 12735 |
|  | R-squared | .114 | .097 | .116 | .128 | .142 |
|  |  |  |  |  |  |  |
|  | *** p<0.01, ** p<0.05, * p<0.1 | | |  |  |  |

Controlling for age and gender, age p<.01 for 2017-2021; gender p<01 for 2017-2020

**S4 Table. Predictors of zero total medical and surgical cross-sectional costs for adults for each year 2017-2021. Two-part regression that models zero cost in 2017 – 2021.**

|  |  | **Zero medical and surgical** | **Zero medical and surgical** | **Zero medical and surgical** | **Zero medical and surgical** | **Zero medical and surgical** |
| --- | --- | --- | --- | --- | --- | --- |
|  |  | **Cost 2017** | **Cost 2018** | **Cost 2019** | **Cost 2020** | **Cost 2021** |
|  |  |  |  |  |  |  |
|  | CCHA 2017 | .152+.025*** |  |  |  |  |
|  |  |  |  |  |  |  |
|  | CCHA 2018 |  | .256+-.027*** |  |  |  |
|  |  |  |  |  |  |  |
|  | CCHA 2019 |  |  | .175+-.022*** |  |  |
|  |  |  |  |  |  |  |
|  | CCHA 2020 |  |  |  | .172+.022*** |  |
|  |  |  |  |  |  |  |
|  | CCHA 2021 |  |  |  |  | .182+.023*** |
|  |  |  |  |  |  |  |
|  |  |  |  |  |  |  |
|  | Observations | 10,331 | 10,604 | 11,042 | 11485 | 12735 |
|  | R-squared | .114 | .097 | .116 | .128 | .142 |
|  |  |  |  |  |  |  |
|  | *** p<0.01, ** p<0.05, * p<0.1 | | |  |  |  |

Controlling for age and gender, age p<.01 for 2017-2021; gender p<01 for 2018,2019, 2021 and p<.05 for 2021

**S5 Table. Predictors of log_10_ total medical cross-sectional costs for adults in each year 2017-2021.**

|  |  |  |  |  |  |  |
| --- | --- | --- | --- | --- | --- | --- |
|  |  | **Total medical** | **Total medical** | **Total medical** | **Total medical** | **Total medical** |
|  |  | **Cost 2017** | **Cost 2018** | **Cost 2019** | **Cost** | **Cost 2021** |
|  |  |  |  |  |  |  |
|  | CCHA2017 | .297+-.011*** |  |  |  |  |
|  |  |  |  |  |  |  |
|  | CCHA2018 |  | .299+-.012*** |  |  |  |
|  |  |  |  |  |  |  |
|  | CCHA2019 |  |  | .308+-.011*** |  |  |
|  |  |  |  |  |  |  |
|  | CCHA2020 |  |  |  | .364+-.012*** |  |
|  |  |  |  |  |  |  |
|  | CCHA2021 |  |  |  |  | .383+-.011*** |
|  |  |  |  |  |  |  |
|  |  |  |  |  |  |  |
|  | Observations | 10,128 | 10,412 | 10,832 | 11,326 | 12,535 |
|  | R-squared | .111 | .101 | .108 | .124 | .133 |
|  |  |  |  |  |  |  |
|  | *** p<0.01, ** p<0.05, * p<0.1Controlling for age and gender, age p<.01 for 2017-2021; gender <01 for 2017-2021for 2017-2021 | | |  |  |  |

**S6 Table. Predictors of zero medical (only) costs for adults cross-sectionally for each year 2017-2021**. **Two-part regression for zero adult total medical costs (only) in 2017-2021.**

|  |  |  |  |  |  |  |
| --- | --- | --- | --- | --- | --- | --- |
|  |  | **Zero medical** | **Zero medical** | **Zero medical** | **Zero medical** | **Zero medical** |
|  |  | **Cost 2017** | **Cost 2018** | **Cost 2019** | **Cost 2020** | **Cost 2021** |
|  | CCHA 2017 | .144+.026*** |  |  |  |  |
|  |  |  |  |  |  |  |
|  | CCHA 2018 |  | .243+.028*** |  |  |  |
|  |  |  |  |  |  |  |
|  | CCHA 2019 |  |  | .185+.024*** |  |  |
|  |  |  |  |  |  |  |
|  | CCHA 2020 |  |  |  | .165+.023*** |  |
|  |  |  |  |  |  |  |
|  | CCHA 2021 |  |  |  |  | .175+.024*** |
|  |  |  |  |  |  |  |
|  |  |  |  |  |  |  |
|  | Observations | 10,128 | 10,412 | 10,832 | 11,326 | 12,535 |
|  | R-squared | .111 | .101 | .108 | .124 | .133 |
|  |  |  |  |  |  |  |
|  | *** p<0.01, ** p<0.05, * p<0.1 | | |  |  |  |

Controlling for age and gender, age p<.01 for 2017-2021; gender <01 for 2018 and 2019 only.

**S7 Table. Predictors of log_10_ total cross-sectional costs for children in each year 2017-** **2021, eliminating newborns.**

|  |  | **Total cost** | **Total cost** | **Total cost** | **Total cost** | **Total cost** |
| --- | --- | --- | --- | --- | --- | --- |
|  |  | **2017** | **2018** | **2019** | **2020** | **2021** |
|  |  |  |  |  |  |  |
|  | CCHA2017 | .603+-.035*** |  |  |  |  |
|  |  |  |  |  |  |  |
|  | CCHA2018 |  | .606+-.037*** |  |  |  |
|  |  |  |  |  |  |  |
|  | CCHA2019 |  |  | .669+-.031*** |  |  |
|  |  |  |  |  |  |  |
|  | CCHA2020 |  |  |  | .592+-.034*** |  |
|  |  |  |  |  |  |  |
|  | CCHA2021 |  |  |  |  | .557+-.028*** |
|  |  |  |  |  |  |  |
|  |  |  |  |  |  |  |
|  | Observations | 3,663 | 3,894 | 4,192 | 4,331 | 4,706 |
|  | R-squared | .117 | .098 | .127 | .082 | .091 |
|  |  |  |  |  |  |  |
|  | *** p<0.01, ** p<0.05, * p<0.1 | | |  |  |  |

Controlling for age and gender, age p<.01 for 2017-2021; gender <01 for 2019 only.

**S8 Table. Predictors of zero cross-sectional costs for children for each year 2017-2021, eliminating newborns. Two-part regression model of zero child total costs that were zero in 2017-2021.**

|  |  | **Zero cost** | **Zero cost** | **Zero cost** | **Zero cost** | **Zero cost** |
| --- | --- | --- | --- | --- | --- | --- |
|  |  | **2017** | **2018** | **2019** | **2020** | **2021** |
|  |  |  |  |  |  |  |
|  | CCHA 2017 | .274+.155* |  |  |  |  |
|  |  |  |  |  |  |  |
|  | CCHA 2018 |  | .037+.064 |  |  |  |
|  |  |  |  |  |  |  |
|  | CCHA 2019 |  |  | .288+.126** |  |  |
|  |  |  |  |  |  |  |
|  | CCHA2020 |  |  |  | .247+.124** |  |
|  | CCHA 2021 |  |  |  |  | .154+.073** |
|  |  |  |  |  |  |  |
|  |  |  |  |  |  |  |
|  | Observations | 3,663 | 3,894 | 4,192 | 4,331 | 4,706 |
|  | R-squared | .117 | .098 | .127 | .082 | .091 |
|  |  |  |  |  |  |  |
|  | *** p<0.001, ** p<0.05, * p<0.1 | | |  |  |  |

Controlling for age and gender, age p<.01 for 2018, and p<.05 for 2019,2020, and 2021; gender NS for ally.

**S9 Table. Predictors of adult admissions cross-sectionally 2017-2021, excluding obstetrics.**

|  |  |  |  |  |  |  |
| --- | --- | --- | --- | --- | --- | --- |
|  |  | **Adult Admissions 2017** | **Adult Admissions 2018** | **Adult Admissions 2019** | **Adult Admissions 2020** | **Adult Admissions 2021** |
|  |  |  |  |  |  |  |
|  |  |  |  |  |  |  |
|  | CCHA2017 | .509+-.021*** |  |  |  |  |
|  |  |  |  |  |  |  |
|  | CCHA2018 |  | .525+-.024*** |  |  |  |
|  |  |  |  |  |  |  |
|  | CCHA2019 |  |  | .518+-.020*** |  |  |
|  |  |  |  |  |  |  |
|  | CCHA2020 |  |  |  | .550+-.022*** |  |
|  |  |  |  |  |  |  |
|  | CCHA2021 |  |  |  |  | .544+-.020*** |
|  |  |  |  |  |  |  |
|  |  |  |  |  |  |  |
|  | Observations | 10,605 | 10,854 | 11,340 | 11,780 | 12,735 |
|  |  |  |  |  |  |  |
|  |  |  |  |  |  |  |
|  | *** p<0.01, ** p<0.05, * p<0.1 | | |  |  |  |

Controlling for age and gender, age p<.01 for 2017, 2019, and p<.05 for 2020; gender P<>01 for 2018, and 2019 and p<.05 for 2021.

|  |  | |  | |  | |  | |  |  |
| --- | --- | --- | --- | --- | --- | --- | --- | --- | --- | --- |
|  |  | | **Child**  **admissions**  **2017** | | **Child admissions 2018** | | **Child admissions 2019** | | **Child admissions 2020** | **Child admissions 2021** |
|  |  | |  | |  | |  | |  |  |
|  | CCHA2017 | | .529+-.059*** | |  | |  | |  |  |
|  |  | |  | |  | |  | |  |  |
|  | CCHA2018 | |  | | .610+-.063*** | |  | |  |  |
|  |  | |  | |  | |  | |  |  |
|  | CCHA2019 | |  | |  | | .675+-.047*** | |  |  |
|  |  | |  | |  | |  | |  |  |
|  | CCHA2020 | |  | |  | |  | | .507+-.054*** |  |
|  |  | |  | |  | |  | |  |  |
|  | CCHA2021 | |  | |  | |  | |  | .462+-.049*** |
|  |  | |  | |  | |  | |  |  |
|  |  | |  | |  | |  | |  |  |
|  | Observations | | 3,663 | | 3,895 | | 4,199 | | 4,333 | 4,706 |
|  |  | |  | |  | |  | |  |  |
|  |  | |  | |  | |  | |  |  |
|  |  |  | |  | |  | |  |  |  |

**S10 Table. Predictors of child admissions in 2017-2021 cross-sectionally, excluding newborns**.

Controlling for age and gender, age p<.01 for 2017-2021; gender NS for 2017-2021.

**S11 Table. Generalized Structural Equation Model (GSEM) estimates the direct effect of comorbidity on total healthcare costs from 2017 to 2021.**

|  |  |  |  |  |  |
| --- | --- | --- | --- | --- | --- |
|  | **2017** | **2018** | **2019** | **2020** | **2021** |
| CCHA 2017 | .518+.021*** |  |  |  |  |
|  |  |  |  |  |  |
| CCHA 2018 |  | .532+.023*** |  |  |  |
|  |  |  |  |  |  |
| CCHA 2019 |  |  | .524+.021*** |  |  |
|  |  |  |  |  |  |
| CCHA 2020 |  |  |  | .557+.021*** |  |
|  |  |  |  |  |  |
| CCHA 2021 |  |  |  |  | .548+.019*** |
|  |  |  |  |  |  |
|  |  |  |  |  |  |
| Observations | 10,705.000 | 10,961.000 | 11,370.000 | 11,874.000 | 12,809.000 |
|  |  |  |  |  |  |
| -2LL | (11,919.000) | (12,885.000) | (13,008.000) | (13,303.000) | (15,111.000) |
| AIC | 23,860.000 | 25,793.000 | 26,038.000 | 26,627.000 | 30,244.000 |
|  |  |  |  |  |  |

*** p<0.01, ** p<0.05, * p<0.1

Controlling for age and gender, age p<.01 for 2017-2021; gender p<01 for 2018,2019,2020 and p<.05 for 2017.

**S12 Table. GSEM estimates of the indirect effect of comorbidity on** **the log_10_ transformed total healthcare costs from 2017 to 2021 mediated by hospital admissions.**

|  |  |  |  |  |  |
| --- | --- | --- | --- | --- | --- |
|  | **2017** | **2018** | **2019** | **2020** | **2021** |
| CCHA 2017 | .124+.005*** |  |  |  |  |
|  |  |  |  |  |  |
| 2017 admit | .307+.018*** |  |  |  |  |
| CCHA 2018 |  | .124+.005*** |  |  |  |
|  |  |  |  |  |  |
| 2018 admit |  | .383+.022*** |  |  |  |
| CCHA 2019 |  |  | .131+.005*** |  |  |
|  |  |  |  |  |  |
| 2019 admit |  |  | .380+.021*** |  |  |
| CCHA 2020 |  |  |  | -.154+.005*** |  |
| 2020 admit |  |  |  | .336+.022*** |  |
| CCHA 2021 |  |  |  |  | .162+.005*** |
| 2021 admit |  |  |  |  | .349+.022*** |
| Observations | 10,705.000 | 10,961.000 | 11,370.000 | 11,874.000 | 12,809.000 |
|  |  |  |  |  |  |
| -2LL | (11,919.000) | (12,885.000) | (13,008.000) | (13,303.000) | (15,111.000) |
| AIC | 23,860.000 | 25,793.000 | 26,038.000 | 26,627.000 | 30,244.000 |

*** p<0.01, ** p<0.05, * p<0.1

Controlling for age and gender, age p<.01 for 2017-2021; gender p<01 for 2017-2021.
