# Supplementary material for: Charlson comorbidity health analytics: A population management strategy to identify risk of hospitalizations, repeated hospitalizations, and resultant high cost
Source: PLoS One. 2026 Jun 29;21(6):e0351956. doi: 10.1371/journal.pone.0351956 (PMC13313358; doi:10.1371/journal.pone.0351956)
Supplement: S7 Table — (DOCX) [file pone.0351956.s007.docx]

**S7 Table. Predictors of log_10_ total cross-sectional costs for children in each year 2017-** **2021, eliminating newborns.**

|  |  | **Total cost** | **Total cost** | **Total cost** | **Total cost** | **Total cost** |
| --- | --- | --- | --- | --- | --- | --- |
|  |  | **2017** | **2018** | **2019** | **2020** | **2021** |
|  |  |  |  |  |  |  |
|  | CCHA2017 | .603+-.035*** |  |  |  |  |
|  |  |  |  |  |  |  |
|  | CCHA2018 |  | .606+-.037*** |  |  |  |
|  |  |  |  |  |  |  |
|  | CCHA2019 |  |  | .669+-.031*** |  |  |
|  |  |  |  |  |  |  |
|  | CCHA2020 |  |  |  | .592+-.034*** |  |
|  |  |  |  |  |  |  |
|  | CCHA2021 |  |  |  |  | .557+-.028*** |
|  |  |  |  |  |  |  |
|  |  |  |  |  |  |  |
|  | Observations | 3,663 | 3,894 | 4,192 | 4,331 | 4,706 |
|  | R-squared | .117 | .098 | .127 | .082 | .091 |
|  |  |  |  |  |  |  |
|  | *** p<0.01, ** p<0.05, * p<0.1 | | |  |  |  |

Controlling for age and gender, age p<.01 for 2017-2021; gender <01 for 2019 only.
