# Supplementary material for: Charlson comorbidity health analytics: A population management strategy to identify risk of hospitalizations, repeated hospitalizations, and resultant high cost
Source: PLoS One. 2026 Jun 29;21(6):e0351956. doi: 10.1371/journal.pone.0351956 (PMC13313358; doi:10.1371/journal.pone.0351956)
Supplement: S8 Table — Two-part regression model of zero child total costs that were zero in 2017–2021. (DOCX) [file pone.0351956.s008.docx]

**S8 Table. Predictors of zero cross-sectional costs for children for each year 2017-2021, eliminating newborns. Two-part regression model of zero child total costs that were zero in 2017-2021.**

|  |  | **Zero cost** | **Zero cost** | **Zero cost** | **Zero cost** | **Zero cost** |
| --- | --- | --- | --- | --- | --- | --- |
|  |  | **2017** | **2018** | **2019** | **2020** | **2021** |
|  |  |  |  |  |  |  |
|  | CCHA 2017 | .274+.155* |  |  |  |  |
|  |  |  |  |  |  |  |
|  | CCHA 2018 |  | .037+.064 |  |  |  |
|  |  |  |  |  |  |  |
|  | CCHA 2019 |  |  | .288+.126** |  |  |
|  |  |  |  |  |  |  |
|  | CCHA2020 |  |  |  | .247+.124** |  |
|  | CCHA 2021 |  |  |  |  | .154+.073** |
|  |  |  |  |  |  |  |
|  |  |  |  |  |  |  |
|  | Observations | 3,663 | 3,894 | 4,192 | 4,331 | 4,706 |
|  | R-squared | .117 | .098 | .127 | .082 | .091 |
|  |  |  |  |  |  |  |
|  | *** p<0.001, ** p<0.05, * p<0.1 | | |  |  |  |

Controlling for age and gender, age p<.01 for 2018, and p<.05 for 2019,2020, and 2021; gender NS for ally.
