# Supplementary material for: Charlson comorbidity health analytics: A population management strategy to identify risk of hospitalizations, repeated hospitalizations, and resultant high cost
Source: PLoS One. 2026 Jun 29;21(6):e0351956. doi: 10.1371/journal.pone.0351956 (PMC13313358; doi:10.1371/journal.pone.0351956)
Supplement: S9 Table — (DOCX) [file pone.0351956.s009.docx]

**S9 Table. Predictors of adult admissions cross-sectionally 2017-2021, excluding obstetrics.**

|  |  |  |  |  |  |  |
| --- | --- | --- | --- | --- | --- | --- |
|  |  | **Adult Admissions 2017** | **Adult Admissions 2018** | **Adult Admissions 2019** | **Adult Admissions 2020** | **Adult Admissions 2021** |
|  |  |  |  |  |  |  |
|  |  |  |  |  |  |  |
|  | CCHA2017 | .509+-.021*** |  |  |  |  |
|  |  |  |  |  |  |  |
|  | CCHA2018 |  | .525+-.024*** |  |  |  |
|  |  |  |  |  |  |  |
|  | CCHA2019 |  |  | .518+-.020*** |  |  |
|  |  |  |  |  |  |  |
|  | CCHA2020 |  |  |  | .550+-.022*** |  |
|  |  |  |  |  |  |  |
|  | CCHA2021 |  |  |  |  | .544+-.020*** |
|  |  |  |  |  |  |  |
|  |  |  |  |  |  |  |
|  | Observations | 10,605 | 10,854 | 11,340 | 11,780 | 12,735 |
|  |  |  |  |  |  |  |
|  |  |  |  |  |  |  |
|  | *** p<0.01, ** p<0.05, * p<0.1 | | |  |  |  |

Controlling for age and gender, age p<.01 for 2017, 2019, and p<.05 for 2020; gender P<>01 for 2018, and 2019 and p<.05 for 2021.
