# Supplementary material for: Charlson comorbidity health analytics: A population management strategy to identify risk of hospitalizations, repeated hospitalizations, and resultant high cost
Source: PLoS One. 2026 Jun 29;21(6):e0351956. doi: 10.1371/journal.pone.0351956 (PMC13313358; doi:10.1371/journal.pone.0351956)
Supplement: S10 Table — (DOCX) [file pone.0351956.s010.docx]

**S10 Table. Predictors of child admissions in 2017-2021 cross-sectionally, excluding newborns**

|  |  | |  | |  | |  | |  |  |
| --- | --- | --- | --- | --- | --- | --- | --- | --- | --- | --- |
|  |  | | **Child**  **admissions**  **2017** | | **Child admissions 2018** | | **Child admissions 2019** | | **Child admissions 2020** | **Child admissions 2021** |
|  |  | |  | |  | |  | |  |  |
|  | CCHA2017 | | .529+-.059*** | |  | |  | |  |  |
|  |  | |  | |  | |  | |  |  |
|  | CCHA2018 | |  | | .610+-.063*** | |  | |  |  |
|  |  | |  | |  | |  | |  |  |
|  | CCHA2019 | |  | |  | | .675+-.047*** | |  |  |
|  |  | |  | |  | |  | |  |  |
|  | CCHA2020 | |  | |  | |  | | .507+-.054*** |  |
|  |  | |  | |  | |  | |  |  |
|  | CCHA2021 | |  | |  | |  | |  | .462+-.049*** |
|  |  | |  | |  | |  | |  |  |
|  |  | |  | |  | |  | |  |  |
|  | Observations | | 3,663 | | 3,895 | | 4,199 | | 4,333 | 4,706 |
|  |  | |  | |  | |  | |  |  |
|  |  | |  | |  | |  | |  |  |
|  |  |  | |  | |  | |  |  |  |

Controlling for age and gender, age p<.01 for 2017-2021; gender NS for 2017-2021.
