# Supplementary material for: Charlson comorbidity health analytics: A population management strategy to identify risk of hospitalizations, repeated hospitalizations, and resultant high cost
Source: PLoS One. 2026 Jun 29;21(6):e0351956. doi: 10.1371/journal.pone.0351956 (PMC13313358; doi:10.1371/journal.pone.0351956)
Supplement: S11 Table — (DOCX) [file pone.0351956.s011.docx]

**S11 Table. Generalized Structural Equation Model (GSEM) estimates the direct effect of comorbidity on total healthcare costs from 2017 to 2021.**

|  |  |  |  |  |  |
| --- | --- | --- | --- | --- | --- |
|  | **2017** | **2018** | **2019** | **2020** | **2021** |
| CCHA 2017 | .518+.021*** |  |  |  |  |
|  |  |  |  |  |  |
| CCHA 2018 |  | .532+.023*** |  |  |  |
|  |  |  |  |  |  |
| CCHA 2019 |  |  | .524+.021*** |  |  |
|  |  |  |  |  |  |
| CCHA 2020 |  |  |  | .557+.021*** |  |
|  |  |  |  |  |  |
| CCHA 2021 |  |  |  |  | .548+.019*** |
|  |  |  |  |  |  |
|  |  |  |  |  |  |
| Observations | 10,705.000 | 10,961.000 | 11,370.000 | 11,874.000 | 12,809.000 |
|  |  |  |  |  |  |
| -2LL | (11,919.000) | (12,885.000) | (13,008.000) | (13,303.000) | (15,111.000) |
| AIC | 23,860.000 | 25,793.000 | 26,038.000 | 26,627.000 | 30,244.000 |
|  |  |  |  |  |  |

*** p<0.01, ** p<0.05, * p<0.1

Controlling for age and gender, age p<.01 for 2017-2021; gender p<01 for 2018,2019,2020 and p<.05 for 2017.
